# Using haplotypes for the prediction of allelic identity to fine-map QTL: characterization and properties

**DOI:** 10.1186/1297-9686-46-45

**Published:** 2014-07-14

**Authors:** Laval Jacquin, Jean-Michel Elsen, Hélène Gilbert

**Affiliations:** 1 INRA, GenPhySE (Génétique, Physiologie et Systèmes d’Elevage), F-31326, Castanet-Tolosan, France; 2 Université de Toulouse, INP, ENSAT, GenPhySE (Génétique, Physiologie et Systèmes d’Elevage), F-31326, Castanet-Tolosan, France; 3 Université de Toulouse, INP, ENVT, GenPhySE (Génétique, Physiologie et Systèmes d’Elevage), F-31076, Toulouse, France

## Abstract

**Background:**

Numerous methods have been developed over the last decade to predict allelic identity at unobserved loci between pairs of chromosome segments along the genome. These loci are often unobserved positions tested for the presence of quantitative trait loci (QTL). The main objective of this study was to understand from a theoretical standpoint the relation between linkage disequilibrium (LD) and allelic identity prediction when using haplotypes for fine mapping of QTL. In addition, six allelic identity predictors (AIP) were also compared in this study to determine which one performed best in theory and application.

**Results:**

A criterion based on a simple measure of matrix distance was used to study the relation between LD and allelic identity prediction when using haplotypes. The consistency of this criterion with the accuracy of QTL localization, another criterion commonly used to compare AIP, was evaluated on a set of real chromosomes. For this set of chromosomes, the criterion was consistent with the mapping accuracy of a simulated QTL with either low or high effect. As measured by the matrix distance, the best AIP for QTL mapping were those that best captured LD between a tested position and a QTL. Moreover the matrix distance between a tested position and a QTL was shown to decrease for some AIP when LD increased. However, the matrix distance for AIP with continuous predictions in the [0,1] interval was algebraically proven to decrease less rapidly up to a lower bound with increasing LD in the simplest situations, than the discrete predictor based on identity by state between haplotypes (IBS _hap_), for which there was no lower bound. The expected LD between haplotypes at a tested position and alleles at a QTL is a quantity that increases naturally when the tested position gets closer to the QTL. This behavior was demonstrated with pig and unrelated human chromosomes.

**Conclusions:**

When the density of markers is high, and therefore LD between adjacent loci can be assumed to be high, the discrete predictor IBS _hap_ is recommended since it predicts allele identity correctly when taking LD into account.

## Background

Numerous methods have been developed to predict allelic identity at an unobserved locus between pairs of chromosome segments. Such predictions are generally carried out by observing allelic similarities between the pairs of chromosome segments that surround this locus [1-3]. It is assumed that chromosome segments that exhibit more similarities have a higher chance of harboring the same allele(s) at this locus. Many of these methods [1-5] use either directly or implicitly the concept of identity-by-descent (IBD), and therefore predict allelic identity based on allelic likeness. Such predictions of allelic identity can be either continuous or discrete in the [0,1] interval. The matrices that contain these predictions for pairs of chromosome segments, at an unobserved locus, can be used in a statistical procedure to detect association between the locus and some phenotypes of interest. For example, these matrices can be interpreted as being proportional to the covariance matrices of the effect of the locus on phenotypes of interest [1,4,6] and therefore play a central role in the statistical analysis of the variability. The similarity between chromosome segments can be measured based on the haplotypes of markers carried by the segments. Indeed, it has been shown that haplotype-based methods have a higher potential to detect trait-marker associations than single-marker methods in some cases [7-16]. Different methods for predicting allelic identity, hereafter named Allelic Identity Predictors (AIP), have been proposed and in this study, we have compared some of these methods i.e.: (1) the probability measure described by Meuwissen and Goddard [1] is the conditional probability of being IBD at an unobserved locus for pairs of haplotypes, given the identical-by-state (IBS) status of alleles spanning that position; (2) the similarity score of Li and Jiang [2] calculates the sum of the number of shared alleles and the length of the longest shared substring that spans an unobserved locus for pairs of haplotypes; (3) the probability model of Browning [3] is based on Variable Length Markov Chains (VLMC) and performs chromosome clustering at a given marker, and in this model, chromosomes that belong to a given cluster are considered as potentially harboring the same unobserved allele(s) locally; and (4) the IBS status of all marker alleles between pairs of haplotypes and (5) the IBS status of single markers alleles, which are the simplest AIP.

In some association studies, such as those that use random effect models for example, the only input that differs from one AIP to another is the similarity (covariance) matrix built for the tested location. Thus, investigating the properties of similarity matrices is another strategy when comparing AIP, since this comparison is generally based on the accuracy of quantitative trait locus (QTL) localization (e.g. root mean square error). The main objective of the present study was to understand the relation between linkage disequilibrium (LD) and allelic identity prediction when using haplotypes, by identifying the properties of similarity matrices in the neighborhood of a QTL and at the QTL. This was performed using a simple distance measure between these matrices and the similarity matrix at the QTL based on the observed allelic identity (IBS). This distance measure was expressed analytically in terms of LD coefficients. There has been an increasing interest in taking advantage of LD for fine-mapping of complex disease genes [17-20] and QTL [21-24]. Nevertheless, to the best of our knowledge, no study has yet used analytical methods to compare AIP in relation to LD. Here, we define a new criterion based on the chosen matrix distance measure, which allows discrimination between the six AIP. We evaluated the consistency of this criterion with the mapping accuracy of the six AIP for a QTL simulated according to different LD patterns and populations.

The simulations were based on two population types, a set of human chromosomes and a set of porcine chromosomes, with different LD and density patterns. In each case, the QTL was a hidden SNP that simulated a biallelic QTL, as previously proposed [4,8,23,25]. Hence, the present study was framed around the common idea that there is a favorable allele at the QTL, which affects an observed trait. In this context, the aim of AIP is to predict, at the QTL, whether both chromosomal segments of any pair harbor the same unobserved favorable allele or not, which is the same as predicting the IBS or non-IBS state of the alleles. A new (6) unreferenced AIP, named trained predictor and abbreviated as TP, is also compared in this paper. This new predictor, based on a matrix distance concept similar to the one used to discriminate between the AIP, performs least squares prediction in a global fashion over chromosomes. The purpose of this predictor was to investigate the behavior of an AIP which performs global training over the chromosomes in relation to local patterns of LD.

## Methods

### Matrix distance comparison

**Figure 1:**
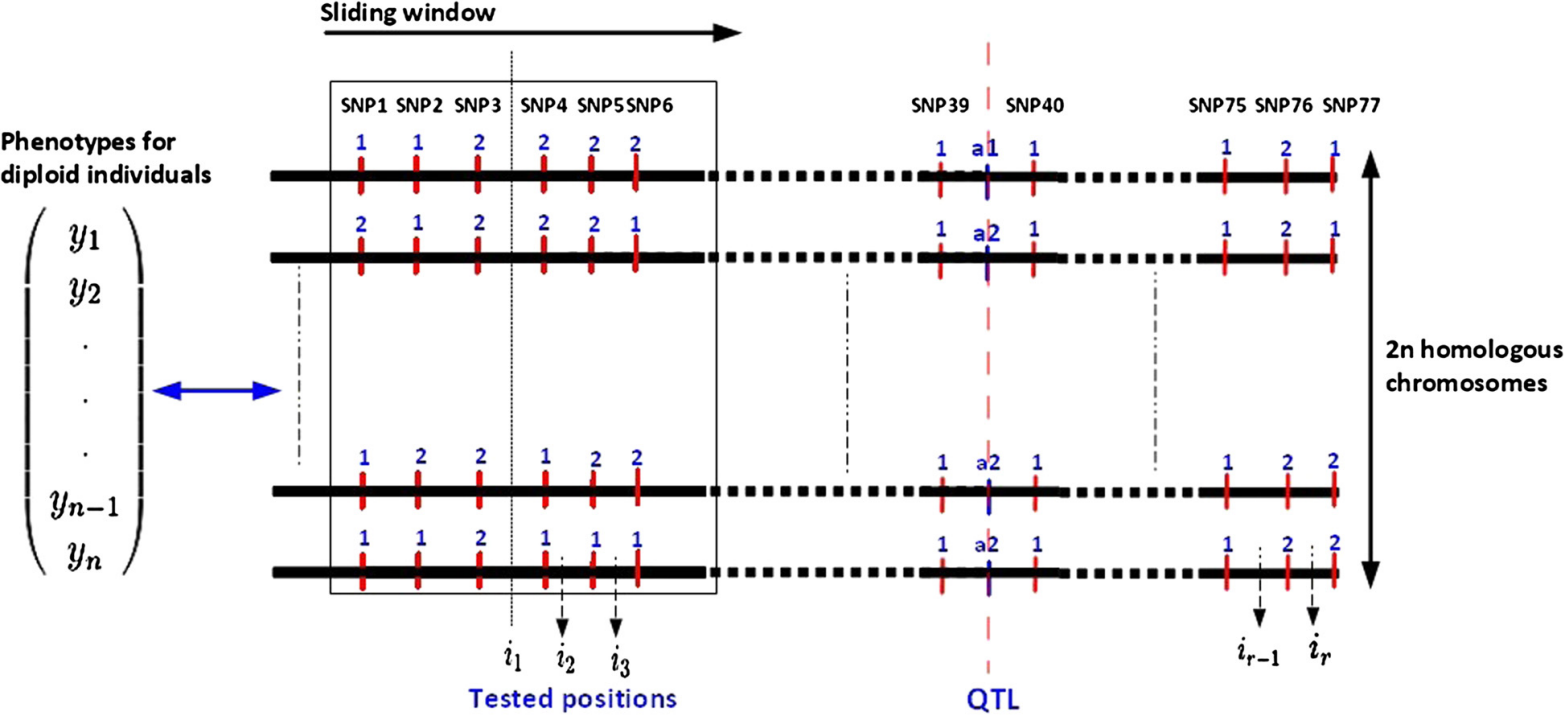
**Principle of the sliding window approach when screening a chromosome for the set *{i***_***1***_ ***,…,ir} *** **of tested positions for associations between phenotypes and 6-marker haplotypes.**

Let $I=\{i_{1},\ldots ,i_{r}\}$ be a set of positions that are tested for the presence of a QTL on 2*n* phased homologous chromosomes for *n* diploid individuals. Only one QTL is considered to be in the screened region. In a sliding window approach, each position tested is considered to be the unobserved center of the haplotypes carried by different chromosome segments. Figure 1 shows an example of tested positions for a sliding window of six markers and a QTL located between SNPs 39 and 40.

Let $s_{i,c_{1},c_{2}}^{P}\in \;[\;0,1]$ be the IBS or IBD prediction of allelic identity, depending on an AIP  at a tested position i∈I, for a couple (*c*_1_,*c*_2_) of chromosome segments. Note that $s_{i,c_{1},c_{2}}^{P}$ is calculated according to the observed similarity between the haplotypes carried by *c*_1_ and *c*_2_. Hence, *c*_1_ and *c*_2_ can harbor different unobserved alleles at *i* even if these segments carry the same haplotype. We define ${\text{M}}^{P,i}={\left(s_{i,c_{1},c_{2}}^{P}\right)}_{1\leq c_{1},c_{2}\leq 2n}$ as the similarity matrix built from the predictions of allelic identity at locus *i* for . Matrix ${\text{M}}^{P,i}$ can be used in a statistical procedure to detect association between *i* and some phenotype of interest.

Let $u_{c_{1},c_{2}}^{\text{QTL}}\in \{0,1\}$ be the true allelic identity observed at the QTL (IBS) for a couple (*c*_1_,*c*_2_) of chromosome segments. On the basis of known alleles at the QTL, the similarity ${\text{M}}^{\text{QTL}}={\left(u_{c_{1},c_{2}}^{\text{QTL}}\right)}_{1\leq c_{1},c_{2}\leq 2n}$ can be built with the real allelic identities. Note that **M**^*QTL*^ is simply a similarity matrix that describes the IBS or non-IBS state of alleles at the QTL.

Let *d*_1_ be a normalized distance measure between ${\text{M}}^{P,i}$ and **M**^*QTL*^ induced by the entrywise 1-norm, which is the sum of the absolute differences between the elements of two matrices or vectors, i.e.

$$\begin{array}{lll} d_{1}\left({\text{M}}^{P,i},{\text{M}}^{\text{QTL}}\right) & =\frac{1}{4n^{2}}∥{\text{M}}^{P,i}-{\text{M}}^{\text{QTL}}{∥}_{1}\; & \;\\ & =\frac{1}{4n^{2}}\overset{2n}{\underset{c_{1}=1}{\sum }}\overset{2n}{\underset{c_{2}=1}{\sum }}|s_{i,c_{1},c_{2}}^{P}-u_{c_{1},c_{2}}^{\text{QTL}}|\; & \; \end{array}$$

Note that some AIP have continuous prediction errors $\left|s_{i,c_{1},c_{2}}^{P}-u_{c_{1},c_{2}}^{\text{QTL}}\right|$ in [ 0,1], while for others, prediction errors are limited to the discrete set {0,1}. Measure *d*_1_ is therefore more appropriate than the euclidean metric *d*_2_, for example, because it does not shrink continuous prediction errors in [ 0,1]. Let *θ*_*QTL*_ be the position of the QTL. When a predictor  performs well, $d_{1}({\text{M}}^{P,i},{\text{M}}^{\text{QTL}})$ should be minimum at the tested position closest to *θ*_*QTL*_. Hence $d_{1}({\text{M}}^{P,i},{\text{M}}^{\text{QTL}})$ can be used to compare different AIP for a set of tested positions. Note that $d_{1}({\text{M}}^{P,i},{\text{M}}^{\text{QTL}})$ can also be expressed as [see Additional file 1]:

(1) $$\begin{array}{ll} \;d_{1}\left({\text{M}}^{P,i},{\text{M}}^{\text{QTL}}\right)= & \overset{K}{\underset{p=1}{\sum }}f_{i,h_{p}}\overset{K}{\underset{q=1}{\sum }}f_{i,h_{q}}\left[\right)p_{i,h_{p},h_{q}}^{\text{QTL}}\times \left(1-s_{i,h_{p},h_{q}}^{P}\right)\\ & \left(+\left(1-p_{i,h_{p},h_{q}}^{\text{QTL}}\right)s_{i,h_{p},h_{q}}^{P}\right] \end{array}$$

where *K*=2^*t*^ is the number of possible observed haplotypes at position *i*, for a sliding window of *t* markers. $f_{i,h_{p}}$ and $f_{i,h_{q}}$ are the frequencies of haplotypes *h*_*p*_ and *h*_*q*_ at position *i*, respectively. Note that some haplotypes among the *K* possible haplotypes may not be observed in practice. Hence, the corresponding frequencies for these haplotypes will naturally be equal to 0 in expression (1). $p_{i,h_{p},h_{q}}^{\text{QTL}}$ is the proportion of identical alleles shared at the QTL by the pairs of chromosomes that carry *h*_*p*_ and *h*_*q*_, at position *i*, and $s_{i,h_{p},h_{q}}^{P}$ is the prediction of allelic identity at locus *i* for the predictor  and a pair (*h*_*p*_,*h*_*q*_) of haplotypes. Expression (1) will be used subsequently to express *d*_1_ as a function of LD coefficients, and to understand the trained predictor defined in this paper.

### Measures of AIP evaluated

The AIP evaluated in this study were IBS _m_ (IBS status of alleles at single markers), IBS _hap_ (IBS status of all marker alleles between pairs of haplotypes), P(IBD) (IBD probability of Meuwissen and Goddard [1]), Score (similarity score of Li and Jiang [2]), Beagle (cluster-based probability model of Browning [3]) and TP (the trained predictor). Note that the tested positions coincide with marker positions for IBS _m_ and Beagle. These positions are therefore different from those in Figure 1. The tested positions for IBS _hap_, P(IBD), Score and TP are defined as presented in Figure 1.

IBS _m_ gives an allelic identity prediction of 1 if a pair of chromosome segments carries the same allele at a tested marker and 0 otherwise. With IBS _hap_ the prediction of allelic identity is equal to 1 if both chromosome segments of a pair carry the same marker alleles for haplotypes that span the tested position *i*, and 0 otherwise. P(IBD) is an estimation of the conditional probability of being IBD at *i* for a pair of chromosome segments, given the IBS status of marker alleles of the haplotypes spanning *i*. This measure of probability is based on a coalescence process and models recombination between markers. The P(IBD) function was applied here with an ancestral effective population size of 100 and 100 generations from the base population, as in Meuwissen and Goddard [1]. Meuwissen and Goddard [23] showed that violations of these assumptions, i.e. that alter the effective population size and the number of generations since the base population, had no effect on the mapping accuracy of their methods [23,26]. For a pair of haplotypes carried by two chromosome segments, Score is the summation of the number of IBS alleles and the length of the longest common substring of IBS alleles that span *i*. Score integrates weight functions that decrease the significance of markers based on their genetic distance from *i*. As proposed in Li and Jiang [2], these functions were chosen to be one minus the distance, in centiMorgan (cM), of each marker from *i* on the haplotypes within the sliding window (as presented in Figure 1). Beagle clusters chromosomes or haplotypes locally at a tested marker if they have similar probabilities of carrying the same alleles at following adjacent markers. The Beagle probability model was built at each marker by running the Beagle software (Beagle 3.3.2; http://faculty.washington.edu/browning/beagle/beagle.html, Browning [3], Browning and Browning [12]) and fitting all the chromosome markers at one time. The Beagle probability model needs two parameters (scale and shift) to be built. These parameters were first estimated from the data using a cross-validation procedure. However, the mapping results were less accurate than those obtained with the default values for these parameters that were proposed by the authors. According to the authors, the default values have performed well in simulation studies and real data analyses [12,27]. Hence the default values scale = 4.0 and shift = 0.2 [12] were used.

The trained predictor (TP), built by least squares prediction, is based on the idea that pairs of haplotypes that exhibit the same amount of allelic similarity should have the same probability of harboring identical alleles, regardless of the tested positions they span. Estimates for ${\left(s_{i,h_{p},h_{q}}^{\text{TP}}\right)}_{(p,q)\in {\left\{1,\mathrm{..},K\right\}}^{2}}$ can be obtained as follows. Let $J=\{j_{1},\ldots ,j_{T}\}$ be a set of observed SNPs on chromosomes, which are called target SNPs. Each target SNP *j* is defined as the middle marker of a sliding window of *t*+1 loci, where *t* is the number of observed flanking markers used to predict allelic identity at the target SNP. Let $u_{j,c_{1},c_{2}}\in \{0,1\}$ be the real allele identity at *j* for (*c*_1_,*c*_2_) and let $E^{\text{TP}}$ be the mean squared prediction errors over  for TP, i.e.

$$\begin{array}{lll} E^{\text{TP}} & =\frac{1}{T}\overset{j_{T}}{\underset{j=j_{1}}{\sum }}\left[\frac{1}{4n^{2}}\overset{2n}{\underset{c_{1}=1}{\sum }}\overset{2n}{\underset{c_{2}=1}{\sum }}{\left(s_{c_{1},c_{2}}^{\text{TP}}-u_{j,c_{1},c_{2}}\right)}^{2}\right]\; & \;\\ & =\frac{1}{T}\overset{j_{T}}{\underset{j=j_{1}}{\sum }}\left[\;d_{2}\left({\text{M}}^{\text{TP},j},{\text{M}}^{j}\right)\;\right]\; & \;\\ & =\frac{1}{T}\overset{j_{T}}{\underset{j=j_{1}}{\sum }}\left[\overset{K}{\underset{p=1}{\sum }}f_{j,h_{p}}\overset{K}{\underset{q=1}{\sum }}f_{j,h_{q}}\left[\;p_{j,h_{p},h_{q}}{\left(s_{h_{p},h_{q}}^{\text{TP}}-1\right)}^{2}\right)\right)\; & \;\\ & \;\left(\left(+\left(1-p_{j,h_{p},h_{q}}\right){\left(s_{h_{p},h_{q}}^{\text{TP}}-0\right)}^{2}\right]\right]\; & \; \end{array}$$

Note that the expression of the normalized squared euclidean distance, *d*_2_, in terms of frequencies and proportions is analogous to that of *d*_1_ in (1).

Indeed $f_{j,h_{p}}$, $f_{j,h_{q}}$ and $p_{j,h_{p},h_{q}}$ at locus *j* are defined as in (1). Estimates for ${\left(s_{i,h_{p},h_{q}}^{\text{TP}}\right)}_{(p,q)\in {\left\{1,\mathrm{..},K\right\}}^{2}}$ are obtained by differentiating $E^{\text{TP}}$ with respect to $s_{h_{p},h_{q}}^{\text{TP}}$, i.e.

$$\frac{\partial E^{\text{TP}}}{\partial s_{h_{p},h_{q}}^{\text{TP}}}=0\Leftrightarrow {ŝ}_{h_{p},h_{q}}^{\text{TP}}=\frac{\overset{j_{T}}{\underset{j=j_{1}}{\sum }}f_{j,h_{p}}f_{j,h_{q}}p_{j,h_{p},h_{q}}}{\overset{j_{T}}{\underset{j=j_{1}}{\sum }}f_{j,h_{p}}f_{j,h_{q}}}$$

Note that the second derivative of $E^{\text{TP}}$ with respect to $s_{h_{p},h_{q}}^{\text{TP}}$ is positive since it is a sum of frequencies. This implies that $E^{\text{TP}}$ reaches a minimum for the set of estimates ${\left({ŝ}_{h_{p},h_{q}}^{\text{TP}}\right)}_{(p,q)\in {\left\{1,\mathrm{..},K\right\}}^{2}}$, since $E^{\text{TP}}$ is a sum of convex functions of each $s_{h_{p},h_{q}}^{\text{TP}}$. Hence, TP associates ${ŝ}_{h_{p},h_{q}}^{\text{TP}}$ to any observed couple (*h*_*p*_,*h*_*q*_) at any tested position i∈I. The observed target SNPs (j∈J) are used to estimate the predictions of allelic identity for TP and should not be confused with the unobserved tested positions (i∈I).

### Statistical models, test statistic and relative efficiency

#### Mixed models

The following mixed models were used to test for the presence of a QTL at a given position i∈I for all AIP:

$$\left\{\begin{array}{ll} Y=X\beta +Z_{h}h+Z_{u}u+\varepsilon & \left({\text{H}}_{1}\right)\\ Y=X\beta +Z_{u}u+\varepsilon & \left({\text{H}}_{0}\right) \end{array}\right)$$

where *β* is a fixed effect, which is the overall mean, and  is a vector of *n* ones. Vector **u** represents the random polygenic effects due to relationships among individuals, i.e. $\text{u}∼N_{n}\left(0,\text{A}\sigma_{\text{u}}^{2}\right)$ where **A** is the additive relationship matrix built from the pedigree [28,29]. **Z**_**h**_ and **Z**_**u**_ are design matrices that link random effects to individuals and ***ε*** is the vector of homoscedastic error terms, i.e. $\varepsilon ∼N_{n}\left(0,{\text{I}}_{n}\sigma_{\varepsilon }^{2}\right)$.

In the model corresponding to (H_1_), **h** is a vector of random effects of haplotypes at position *i*, i.e. $\text{h}∼N_{\kappa }(0,{\text{H}}^{P,i}\sigma_{\text{h}}^{2})$, where *κ* (*κ*≤*K*) corresponds to the number of observed haplotypes, or alleles, at position *i*. Note that **h** has the same dimension *κ* for all AIP except for IBS _m_ and Beagle. The tested positions coincide with marker positions for these two predictors. At a tested marker *i*, *κ*=2 for IBS _m_ and *κ* is equal to the number of local clusters for Beagle. Therefore, depending on the predictor , ${\text{H}}^{P,i}$ is a similarity matrix based on either distinct observed haplotypes (e.g.  Score) or distinct clusters (e.g. P= Beagle). Note that ${\text{H}}^{P,i}$ and ${\text{M}}^{P,i}$ are equivalent sources of data contingent upon the list of haplotypes, or distinct local clusters, for the chromosome segments at any tested position. Indeed, depending on , one can build ${\text{M}}^{P,i}$ from ${\text{H}}^{P,i}$ in one of the two following ways. (1) ${\text{M}}_{c_{1},c_{2}}^{P,i}={\text{H}}_{h(c_{1}),h(c_{2})}^{P,i}$, where *h*(*c*_1_) and *h*(*c*_2_) are the haplotype numbers carried by chromosomes *c*_1_ and *c*_2_, respectively or (2) ${\text{M}}_{c_{1},c_{2}}^{P,i}={\text{H}}_{C(c_{1}),C(c_{2})}^{P,i}$, where $C(c_{1})$ and $C(c_{2})$ are the cluster numbers to which chromosomes *c*_1_ and *c*_2_ belong, respectively.

#### RLRT statistic

The Expectation-Maximization algorithm was used for the restricted maximum likelihoods of the mixed models [30-33], to estimate the components *β*, **h**, **u**, *ε* and the variance terms $\sigma_{\text{h}}^{2},\sigma_{\text{u}}^{2},\sigma_{\varepsilon }^{2}$. Let $\lambda_{i}^{P}$ be the restricted maximum likelihood ratio test (RLRT) of (H_1_) versus (H_0_) for position *i*, i.e.

$$\lambda_{i}^{P}=-2\text{ln}\left(\frac{L_{\text{REML}}({\text{H}}_{0})}{L_{\text{REML}}^{P}({\text{H}}_{1})}\right)$$

We defined $\theta_{\text{m.a.}}^{P}$ as the estimated position of a QTL for a predictor , i.e.

$$\theta_{\text{m.a.}}^{P}=\underset{i\in I}{\text{argmax}}\left\{{\hat{\lambda }}_{i}^{P}\right\}$$

#### Relative efficiency

To compare the predictive ability of the different predictors in relation to LD, we defined $\theta_{\text{r.e.}}^{P}$ as the tested position where $d_{1}\left({\text{M}}^{P,i},{\text{M}}^{\text{QTL}}\right)$ is minimized for a predictor , i.e.

$$\theta_{\text{r.e.}}^{P}=\underset{i\in I}{\text{argmin}}\left\{d_{1}\left({\text{M}}^{P,i},{\text{M}}^{\text{QTL}}\right)\right\}$$

Consequently, we defined the relative efficiency of a predictor  as follows. Predictor  is considered to be more efficient than a predictor $P^{\prime }$ if

$$\left\{\begin{array}{ll} \left|\theta_{\text{r.e.}}^{P}-\theta_{\text{QTL}}|<|\theta_{\text{r.e.}}^{P^{\prime }}-\theta_{\text{QTL}}\right| & \left(a\right)\\ d_{1}\left({\text{M}}^{P,\theta_{\text{r.e.}}^{P}},{\text{M}}^{\text{QTL}}\right)<d_{1}\left({\text{M}}^{P^{\prime },\theta_{\text{r.e.}}^{P^{\prime }}},{\text{M}}^{\text{QTL}}\right) & \left(b\right) \end{array}\right)$$

where |.| is the absolute value. When $\theta_{\text{r.e.}}^{P}$ was not unique, the mean of the different argmins was retained as $\theta_{\text{r.e.}}^{P}$. Inequality (*a*) states that the tested position associated with the best prediction, of the allele identity at the QTL, is closer to the QTL for  than that for $P^{\prime }$. Inequality (*b*) states that the true allelic identity at the QTL is better predicted by  at $\theta_{\text{r.e.}}^{P}$ than by $P^{\prime }$ at $\theta_{\text{r.e.}}^{P^{\prime }}$.

### Comparison criteria

*N* simulations (*w*=1,..,*N*) were performed to evaluate the mapping accuracy and the relative efficiency of the different AIP in different situations.

#### Mapping accuracy

The mapping accuracy of the simulated QTL was evaluated for each AIP with the root mean square error (RMSE):

$${\text{RMSE}}^{\text{m.a.}}=\sqrt{\frac{1}{N}\overset{N}{\underset{w=1}{\sum }}{\left(\theta_{\text{m.a.}}^{P,w}-\theta_{\text{QTL}}\right)}^{2}}$$

#### Relative efficiency

The relative efficiency of each AIP was evaluated by considering the three following quantities:

$$\left\{\begin{array}{l} {\text{RMSE}}^{\text{r.e.}}=\sqrt{\frac{1}{N}\overset{N}{\underset{w=1}{\sum }}{\left(\theta_{\text{r.e.}}^{P,w}-\theta_{\text{QTL}}\right)}^{2}}\\ {\hat{E}}^{\text{r.e.}}=\frac{1}{N}\overset{N}{\underset{w=1}{\sum }}d_{1}\left({\text{M}}^{P,\theta_{\text{r.e.}}^{P,w}},{\text{M}}^{\text{QTL},w}\right)\\ {\hat{\sigma }}^{\text{r.e.}}=\sqrt{\frac{1}{N}\overset{N}{\underset{w=1}{\sum }}{\left(d_{1}\left({\text{M}}^{P,\theta_{\text{r.e.}}^{P,w}},{\text{M}}^{\text{QTL},w}\right)-{\hat{E}}^{\text{r.e.}}\right)}^{2}} \end{array}\right)$$

where RMSE ^r.e.^ and ${\hat{E}}^{\text{r.e.}}$ measure conditions (*a*) and (*b*), defined in the paragraph on relative efficiency, and ${\hat{\sigma }}^{\text{r.e.}}$ measures the standard deviation of the matrix distance at $\theta_{\text{r.e.}}^{P}$.

### Data for simulation

A sliding window of *t*=6 markers was chosen for all analyses, except for IBS _m_ and Beagle. Windows of six and 12 markers were previously shown to be optimal for QTL mapping accuracy [34,35] with 60K type SNP chips. Hence, all analyses were done using a sliding window of *t*=6 markers, except for IBS _m_ and Beagle, to make comparison between the series of results easier. A set of 90 human chromosomes 21 from unrelated Han Chinese individuals from Beijing (HCB), and a set of 235 swine chromosomes 18 from French Large White (FLW) pigs, were used for LD and matrix distance computations. The 90 HCB chromosomes were genotyped for 16 881 SNPs and are available from the HapMap project website (http://hapmap.ncbi.nlm.nih.gov/downloads/phasing/2005-03_{p}haseI/full/). The FLW chromosomes were genotyped for 1252 SNPs using the Illumina Porcine 60K+SNP iSelect Beadchip [36]. Only 14 976 SNPs on the HCB chromosomes and 969 SNPs on the FLW chromosomes for which the minor allele frequency was greater than 5% were retained for analysis. The LD and matrix distance computations were conducted for the HCB and the FLW chromosomes. The QTL simulations were only conducted for the FLW chromosomes for which a pedigree was available. The marker density varied across the FLW chromosomes based on physical distance in kilobase. One megabase was considered equivalent to 1 cM for conversion in this study.

### Variation of LD between tested positions and a QTL

LD between a tested position *i* and a QTL was measured using the multiallelic measure *R* of LD as suggested by [37-39]. Let $\Delta_{p}=f_{i,h_{p}a_{1}}^{\text{QTL}}-f_{i,h_{p}}f_{a_{1}}$ be the LD coefficient between haplotype *h*_*p*_ at position *i* and allele *a*_1_ at the QTL. $f_{i,h_{p}a_{1}}^{\text{QTL}}$ is the frequency of haplotype *h*_*p*_*a*_1_ defined by the marker haplotype *h*_*p*_ that spans position *i* and allele *a*_1_ at the QTL. $f_{a_{1}}$ is the frequency of allele *a*_1_ at the QTL and $f_{i,h_{p}}$ is haplotype *h*_*p*_ frequency at *i*. Note that $-\Delta_{p}=f_{i,h_{p}a_{2}}^{\text{QTL}}-f_{i,h_{p}}f_{a_{2}}$. Hence, for a biallelic QTL, *R* can be expressed as:

$$\begin{array}{lll} R_{i,\text{QTL}} & =\frac{\overset{K}{\underset{p=1}{\sum }}\overset{2}{\underset{l=1}{\sum }}{\left(f_{i,h_{p}a_{l}}^{\text{QTL}}-f_{i,h_{p}}f_{a_{l}}\right)}^{2}}{\left(1-\overset{K}{\underset{p=1}{\sum }}f_{i,h_{p}}^{\text{QTL}\;2}\right)\left(1-\overset{2}{\underset{l=1}{\sum }}f_{a_{l}}^{\;2}\right)}\; & \;\\ & =\frac{\overset{K}{\underset{p=1}{\sum }}\left[{\left(\Delta_{p}\right)}^{2}+{\left(-\Delta_{p}\right)}^{2}\right]}{\left(1-\overset{K}{\underset{p=1}{\sum }}f_{i,h_{p}}^{\text{QTL}\;2}\right)\left(1-\overset{2}{\underset{l=1}{\sum }}f_{a_{l}}^{\;2}\right)}\; & \;\\ & =\frac{2\overset{K}{\underset{p=1}{\sum }}\Delta_{p}^{2}}{\left(1-\overset{K}{\underset{p=1}{\sum }}f_{i,h_{p}}^{\text{QTL}\;2}\right)\left(1-\overset{2}{\underset{l=1}{\sum }}f_{a_{l}}^{\;2}\right)}=\frac{D_{i,\text{QTL}}^{2}}{H_{i}.H_{\text{QTL}}}\; & \; \end{array}$$

where $H_{i}=1-\sum_{p=1}^{K}f_{i,h_{p}}^{\text{QTL}\;2}$ and $H_{\text{QTL}}=1-\sum_{l=1}^{2}f_{a_{l}}^{\;2}$ are the Hardy-Weinberg heterozygosities at *i* and the QTL respectively and $D_{i,\text{QTL}}^{2}=2\sum_{p=1}^{K}\Delta_{p}^{2}$. *R*_*i*,*QTL*_ and $D_{i,\text{QTL}}^{2}$ are expected to increase as the tested position *i* gets closer to a QTL. The general behaviors of the normalized measure *R*_*i*,*QTL*_ and the non-normalized measure $D_{i,\text{QTL}}^{2}$ were described by computing the LD between the haplotypes at successive distinct positions, using a sliding window, and the alleles of a fixed SNP centered over a region of 81 markers on the chromosomes. The fixed SNP was centered over a region of 76 distinct overlapping sliding windows available within the region of 81 markers. The 76 distinct positions associated to the windows played the role of the tested positions of an association study. The fixed SNP played the role of a biallelic QTL. The computation was repeated for all possible regions of 81 successive markers. Since 969 SNPs were retained on the 235 porcine chromosomes, computation was performed for 889 (969-81 + 1 = 889) regions of 81 markers. The same procedure was performed on the HCB chromosomes, thus leading to 14 896 possible regions for this set of chromosomes. The empirical means of the 889 FLW and the 14 896 HCB LD profiles were then computed to describe the expected behaviors of *R*_*i*,*QTL*_ and $D_{i,\text{QTL}}^{2}$. Another major purpose of these computations was to help the analytical comparison of the AIP and the associated matrix distances, which can be expressed as elements of multiallelic LD (see Results section).

### Distributions of matrix distance as a function of multiallelic LD

The distributions of the matrix distance for the six compared AIP, as function of local multiallelic LD, were also evaluated on the FLW and HCB chromosomes. The matrix distances for the six AIP were calculated at 966 and 14 973 possible target SNPs for the FLW and HCB chromosomes, respectively. The target SNPs were defined in exactly the same way as used for the trained predictor (TP). The matrix distances calculated at each window that harbors a target SNP for the six AIP were then plotted against the multiallelic measure *R* of LD between the haplotypes and the target alleles within the window.

### QTL simulation on FLW chromosomes

The 235 FLW chromosomes were included in *N*=200 gene-drop simulations, in a 25-generation pedigree for the FLW breed, using the LDSO software [40]. The pedigree was composed of 1594 founders, 3373 sires and 7100 dams. The gene-drop procedure was used to generate different realistic genealogy structures between the chromosomes. For each gene-drop the 235 FLW chromosomes were uniformly distributed, with replacement, among the 1594 founders of the pedigree. Hence, the measured LD structure for mapping among descendant individuals at the end of each gene-drop was almost the same as on the 235 FLW chromosomes. It must be emphasized that the use of replicates of only 235 chromosomes to populate 1594 diploid founders, followed by 25 generations of recombinations events, means that the number of different haplotypes at a position is much lower than 3188 (2 × 1594). Thus, the results correspond to medium range population sizes. After each gene-drop, only the chromosomes and phenotypes of the *n*=485 individuals of generation 25 were retained for subsequent analyzes.

Three distant SNPs were chosen as putative QTL, in order to have different LD levels with the six-marker haplotype that surrounds them on the 235 initial FLW chromosomes. Two different QTL effects were simulated for each of these SNPs, thus leading to six different scenarios. The LD between these SNPs and the observed haplotypes that harbored them was measured using the multiallelic measure *R* of LD. The LD levels around the three SNPs were equal to 0.52, 0.18 and 0.08, and the lengths of the haplotypes harboring them were equal to 0.09 cM, 0.37 cM and 0.75 cM, respectively. Note that these differences in length were due to the different marker densities in the distinct regions that harbor each putative QTL. The length of the region scanned for QTL mapping around each simulated QTL was approximately 3 cM.

The phenotypes in the pedigree were computed as $y_{i}=\frac{1}{2}\left(p_{i}^{f}+p_{i}^{m}\right)+\phi_{i}+g_{i}^{\text{QTL}}+\delta$, where $p_{i}^{f},p_{i}^{m}$ are normal random polygenic effects of the parents with variance 0.5, *ϕ*_*i*_ is a normal random mendelian sampling effect with variance 0.25 and *δ* is a normal random environmental effect with variance 1. $g_{i}^{\text{QTL}}$ is the QTL genotype effect of individual *i*. QTL genotype effect was first computed as $g_{i}^{\text{QTL}}=2$ or 0 or −2, if the QTL genotype of individual *i* was *a*_1_*a*_1_ or *a*_1_*a*_2_ or *a*_2_*a*_2_ respectively. In the same way a second set of simulations was carried out with the QTL genotype effect computed as $g_{i}^{\text{QTL}}=0.5$ or 0 or −0.5. Only the gene-drop simulations for which the minor allele frequency at the QTL was greater or equal to 0.1 were retained. Each simulated QTL was verified for Hardy-Weinberg equilibrium during simulations. Hence, under the standard model, where the dominance effect is equal to 0 as in this study, the first simulated QTL effect explained at most 57% of the phenotypic variance for equal frequencies at the QTL. In the same way, the second simulated QTL effect explained at most 8% of the phenotypic variance.

## Results

**Figure 2:**
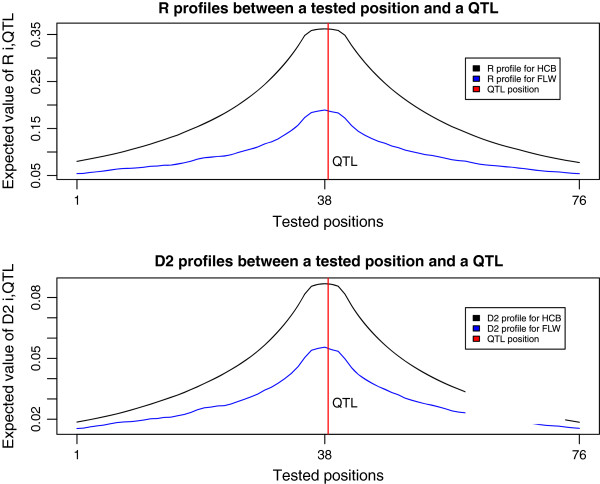
**Empirical means of the 889 FLW and 14 896 HCB LD profiles, obtained for the normalized and the non-normalized multiallelic LD between tested positions (tested position *i *** **= center of six marker haplotypes) and a biallelic QTL (red vertical line), for regions of 81 markers on chromosomes.**

This section gives theoretical and empirical results that show that, compared to others, some AIP exhibit a better behavior for the decrease of their matrix distance, as defined by expression (1), when the multiallelic LD between a tested position and a QTL increases. In summary, the theoretical results show that expression (1) can be written as a function of the multiallelic LD coefficients of *R*, and that the decreasing behavior of this function depends on the nature of the AIP (see equations (2), (3), (4), (5) and (6) of this section). The empirical results show that *R* is expected to be highest when the tested position is closest to the QTL (see Figure 2 of this section). The expectation taken for the multiallelic LD was the empirical mean, which was found to converge for distant regions on the chromosomes. These regions can be assumed to be independent, thus showing an expected behavior for the multiallelic LD. The empirical results also show that the tested position that minimizes the matrix distance is highly correlated with the mapping accuracy of the AIP (see sub-section on mapping accuracy and relative efficiency of this section).

### Variation of LD between tested positions and a QTL

Figure 2 shows the empirical means of the 889 FLW and the 14 896 HCB LD profiles for *R*_*i*,*QTL*_ and $D_{i,\text{QTL}}^{2}$.

In Figure 2 the values of *R*_*i*,*QTL*_ and $D_{i,\text{QTL}}^{2}$ increase, as expected, as the tested position *i* moves closer to the QTL. This implies that the sum of the $\Delta_{p}^{2}$ terms increases on average as position *i* moves toward the QTL. The highest expected values for *R*_*i*,*QTL*_ and $D_{i,\text{QTL}}^{2}$, in Figure 2 are reached for the tested position closest to the QTL. Note that the range of values for $D_{i,\text{QTL}}^{2}$, in Figure 2 is smaller than that of *R*_*i*,*QTL*_. This is due to the lack of a normalization factor for $D_{i,\text{QTL}}^{2}$.

### Matrix distance as function of multiallelic LD coefficients

Based on expression (1), $d_{1}({\text{M}}^{P,i},{\text{M}}^{\text{QTL}})$ can be re-written as [see Additional file 1]:

(2) $$\begin{array}{lll} \;d_{1}\left({\text{M}}^{P,i},{\text{M}}^{\text{QTL}}\right) & =\overset{K}{\underset{p=1}{\sum }}\overset{K}{\underset{q=1}{\sum }}\left[\left[f_{i,h_{p}a_{1}}^{\text{QTL}}f_{i,h_{q}a_{1}}^{\text{QTL}}\right)\right)\; & \;\\ & \;\left(+\;f_{i,h_{p}a_{2}}^{\text{QTL}}f_{i,h_{q}a_{2}}^{\text{QTL}}\right]\left(1-s_{i,h_{p},h_{q}}^{P}\right)\; & \;\\ & \;\left(+\left[f_{i,h_{p}a_{2}}^{\text{QTL}}f_{i,h_{q}a_{1}}^{\text{QTL}}+\;f_{i,h_{p}a_{1}}^{\text{QTL}}f_{i,h_{q}a_{2}}^{\text{QTL}}\right]s_{i,h_{p},h_{q}}^{P}\right]\; \end{array}$$

Replacing the 2*K* frequencies in expression (2) by the (**Δ**_***p***_)_1≤*p*≤*K*_ LD coefficient terms and the product of marginal frequencies gives [see Additional file 1]:

(3) $$\begin{array}{lll} d_{1}\left({\text{M}}^{P,i},{\text{M}}^{\text{QTL}}\right) & =\overset{K}{\underset{p=1}{\sum }}\left[4\left(\overset{K}{\underset{q\neq p}{\sum }}s_{i,h_{p},h_{q}}^{P}-s_{i,h_{p},h_{p}}^{P}\right)\Delta_{p}^{2}\right)\; & \;\\ & \left(\;+\Psi_{\text{pq}}^{P}\left(\Delta_{l\neq p,q}\right)\Delta_{p}+\Phi_{\text{pq}}^{P}\left(\Delta_{l\neq p,q}\right)\right]\; & \;\\ & =\xi^{P}\left(\Delta_{1},\mathrm{..},\Delta_{K}\right), \end{array}$$

where $\Psi_{\text{pq}}^{P}\left(\Delta_{l\neq p,q}\right)$ and $\Phi_{\text{pq}}^{P}\left(\Delta_{l\neq p,q}\right)$ are sums and products of marginal frequencies, allelic identity predictions and LD coefficient terms. The general behavior of $\xi^{P}\left(\Delta_{1},\mathrm{..},\Delta_{K}\right)$, with respect to *R*_*i*,*QTL*_, is unspecifiable due to its complexity. For instance, the behavior of $\xi^{P}\left(\Delta_{1},\mathrm{..},\Delta_{K}\right)$ cannot be specified for continuous AIP in [0,1]. However for , $\xi^{P}\left(\Delta_{1},\mathrm{..},\Delta_{K}\right)$ reduces to a sum of strictly concave functions of each LD coefficient [see Additional file 1], i.e.

(4) $$\begin{array}{lll} \xi^{\text{IBS}{\;}_{\text{hap}}}\left(\Delta_{1},\mathrm{..},\Delta_{K}\right) & =\overset{K}{\underset{p=1}{\sum }}\left[-4\Delta_{p}^{2}+\Psi_{\text{pq}}^{\text{IBS}{\;}_{\text{hap}}}\Delta_{p}\right)\; & \;\\ & \;\left(+\;\Phi_{\text{pq}}^{\text{IBS}{\;}_{\text{hap}}}\right]\; & \;\\ & =\overset{K}{\underset{p=1}{\sum }}\left[Q_{p}(\Delta_{p})\right], \end{array}$$

where $\Psi_{\text{pq}}^{\text{IBS}{\;}_{\text{hap}}}$ and Φ_*pq*_ are sums and products of marginal frequencies that do not depend on LD coefficients. Let $\Delta_{p}^{*}=\frac{\Psi_{\text{pq}}^{\text{IBS}{\;}_{\text{hap}}}}{8}$ be the critical value for each *Q*_*p*_ function. $\xi^{\text{IBS}{\;}_{\text{hap}}}\left(\Delta_{1},\mathrm{..},\Delta_{K}\right)$ will decrease if the squared or absolute deviation of each **Δ**_***p***_ term from its corresponding $\Delta_{p}^{*}$ increases [see Additional file 2]. However note that the squared deviations of all **Δ**_***p***_ terms from their corresponding critical values do not need to increase simultaneously for $\xi^{\text{IBS}{\;}_{\text{hap}}}\left(\Delta_{1},\mathrm{..},\Delta_{K}\right)$ to decrease. For example, some *Q*_*p*_ functions corresponding to haplotypes with low frequencies can be negligible in expression (4). Hence, if $\sum_{p=1}^{K}{\left(\Delta_{p}-\Delta_{p}^{*}\right)}^{2}$ increases sufficiently, $\xi^{\text{IBS}{\;}_{\text{hap}}}\left(\Delta_{1},\mathrm{..},\Delta_{K}\right)$ will decrease. It can be shown that $\sum_{p=1}^{K}{\left(\Delta_{p}-\Delta_{p}^{*}\right)}^{2}$ will increase if $\sum_{p=1}^{K}{\Delta_{p}}^{2}$ increases and that these two quantities share almost the same pattern for their expected values [see Additional file 2]. Thus, according to the $D_{i,\text{QTL}}^{2}$ profiles in Figure 2, $\xi^{\text{IBS}{\;}_{\text{hap}}}\left(\Delta_{1},\mathrm{..},\Delta_{K}\right)$ is expected to decrease as position *i* moves toward the QTL position.

An important result for $\xi^{P}\left(\Delta_{1},\mathrm{..},\Delta_{K}\right)$ is obtained when only two haplotypes are observed among the *K* possible haplotypes. In this case, $\xi^{P}\left(\Delta_{1},\mathrm{..},\Delta_{K}\right)$ reduces to a real function of one LD coefficient [see Additional file 1], i.e.:

(5) $$\begin{array}{lll} \xi^{P}(\Delta_{1}) & =\left[-4s_{i,h_{1},h_{1}}^{P}-4s_{i,h_{2},h_{2}}^{P}+8s_{i,h_{1},h_{2}}^{P}\right]\Delta_{1}^{2}\; & \;\\ & \;+\Psi^{P}\Delta_{1}+\Phi^{P},\; \end{array}$$

where $\Psi^{P}$ and $\Phi^{P}$ are terms independent of LD, and the minimum and maximum possible values for **Δ**_**1**_ are given by $-\frac{1}{4}$ and $\frac{1}{4}$, respectively. If  we have:

(6) $$\begin{array}{ll} \xi^{\text{IBS}{\;}_{\text{hap}}}(\Delta_{1}) & =-8\Delta_{1}^{2}+\Psi^{\text{IBS}{\;}_{\text{hap}}}\Delta_{1}+\Phi^{\text{IBS}{\;}_{\text{hap}}} \end{array}$$

The minimum and maximum possible values for the critical value, $\Delta_{1}^{*}$, of $\xi^{\text{IBS}\;{\;\;\;}_{\text{hap}}}$ are given by $-\frac{1}{4}$ and $\frac{1}{4}$, respectively, if the tested locus and the QTL are monomorphic [see Additional file 1]. In other words, the critical value of this function will always lie within the range of the LD coefficient when LD exist. In expression (5), the coefficient $\left[-4s_{i,h_{1},h_{1}}^{P}-4s_{i,h_{2},h_{2}}^{P}+8s_{i,h_{1},h_{2}}^{P}\right]$ is always greater or equal to −8 for any other predictor  than IBS_hap_, since $s_{i,h_{1},h_{2}}^{P}\in \;[\;0,1]$. For instance, AIP by construction assign positive values to $s_{i,h_{1},h_{2}}^{P}$ when haplotypes *h*_1_ and *h*_2_ share allele similarity. This property is even truer if *h*_1_ and *h*_2_ are very similar. In such cases, the highest rate of decrease for $\xi^{P}$, with respect to the absolute deviation of **Δ**_**1**_ from $\Delta_{1}^{*}$, is thus induced by . Moreover, for such cases, we also have $\xi^{P}\left(-\frac{1}{4}\right)=\xi^{P}\left(\frac{1}{4}\right)\in \left[\frac{1}{2}s_{i,h_{1},h_{2}}^{P},1\right]$, which expresses a lower bound for $\xi^{P}$ (i.e. $\frac{1}{2}s_{i,h_{1},h_{2}}^{P}$, [see Additional file 1]). Finally, $\xi^{P}\left(-\frac{1}{4}\right)=\xi^{P}\left(\frac{1}{4}\right)=0$ if and only if . In other words, when LD between the haplotypes and the QTL alleles is complete, the matrix distance is equal to 0 if and only if  [see Additional file 1]. The decreasing behavior of $\xi^{P}$ between a tested position and a QTL for a substantial increase of LD is therefore deteriorated for AIP with continuous predictions in [ 0,1]. Hence, this result questions the behavior of AIP with continuous predictions in [ 0,1] in relation to LD, in the general case where *K* is greater than 2.

### Distributions of matrix distance as function of multiallelic LD

**Figure 3:**
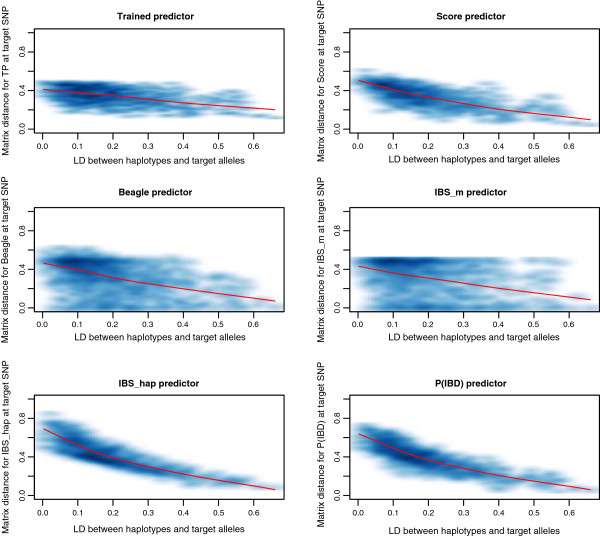
**The matrix distance at 966 target SNPs on the 235 FLW chromosomes for the six compared AIP.** Darker and lighter blue regions correspond to higher and lower density of points. The red lines correspond to LOESS regressions of the matrix distance on the multiallelic LD.

**Figure 4:**
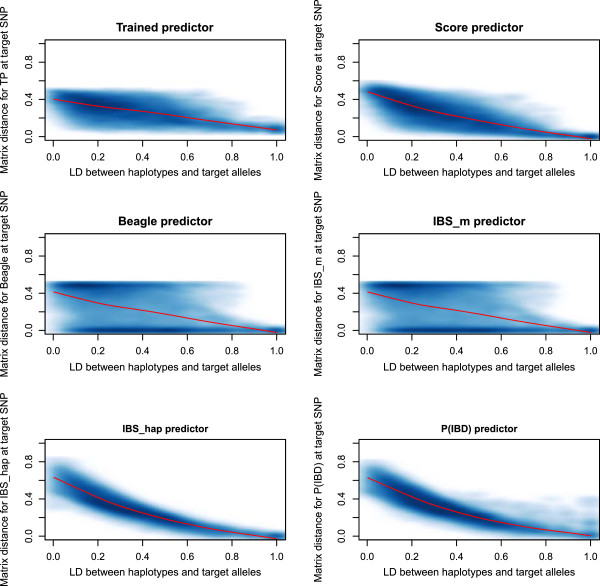
**The matrix distance at 14973 target SNPs on the 90 HCB chromosomes for the six compared AIP.** Darker and lighter blue regions correspond to higher and lower density of points. The red lines correspond to LOESS regressions of the matrix distance on the multiallelic LD.

Figures 3 and 4 show the distributions of the matrix distance for the six AIP against the local multiallelic LD. Figures 3 and 4 convey only local information for the case where the tested position is closest to the QTL, as opposed to Figure 2. Darker and lighter blue regions in Figures 3 and 4 correspond to higher and lower density of points. The red lines in Figures 3 and 4 correspond to non-parametric LOESS regressions of the matrix distance on the multiallelic LD.

Figures 3 and 4 show a better behavior of IBS _hap_ and P(IBD) for the decrease of their matrix distance, with lower variability around the LOESS curves compared to the other predictors, when the LD between the haplotypes and the target alleles increases. The distributions of the matrix distance for IBS _hap_ and P(IBD) in these figures show similar trends on the FLW and HCB chromosomes. This is due to the fact that these two predictors perform similarly in some conditions (see sub-section on mapping accuracy and relative efficiency). However IBS _hap_ shows a better behavior compared to all other predictors in Figures 3 and 4, for the decrease of its matrix distance with increasing multiallelic LD. The good behavior of IBS _hap_ for the decrease of its matrix distance in Figures 3 and 4 is totally explained by equation (4), where the sum of the concave polynomials decreases as the multiallelic LD increases. The better behavior of IBS _hap_, compared to the other predictors in Figures 3 and 4, is explained by equations (3) and (5), which show that continuous predictions in [0,1] will deteriorate the decrease of the matrix distance with respect to LD.

The matrix distances for Beagle and IBS _m_ were also plotted against the local multiallelic LD between the haplotypes and the target alleles in Figures 3 and 4, although these two predictors are defined for marker positions only. Indeed, one of the aims of this study was to compare the AIP based on local LD between haplotypes and alleles at a hidden locus. TP, Score, Beagle and IBS _m_ showed poor relationships for the decrease of their matrix distance with the increasing multiallelic LD. The matrix distance distributions showed high variability for these predictors with respect to *R* on the FLW and HCB chromosomes. Note that the length of the six marker haplotypes on the HCB chromosomes were equal to 0.01 cM, on average, compared to 0.31 cM on average for those on the FLW chromosomes.

### Mapping accuracy and relative efficiency

Table 1 relates the relative efficiency of the six AIP that were compared, and their mapping accuracies, for a QTL simulated under six scenarios on the FLW chromosomes for *N*=200 simulations. $R_{i^{*},\text{QTL}}$ in Table 1 corresponds to the multiallelic LD at position *i*^∗^, measured between the marker-haplotypes that harbor the simulated QTL and the QTL alleles. Note that the tested position *i*^∗^ does not necessarily coincide with the QTL position. Thus, *i*^∗^ can be defined as the tested position closest to the simulated QTL.

**Table 1:** Relative efficiencies and mapping accuracies for different QTL effects

**AIP**				**IBS** ***mQTL***	**IBS**_***m***_	**TP**	**Score**	**IBS**_***hap***_	**P(IBD)**	**Beagle**
$R_{i^{*},\text{QTL}}=0.52$	Relative efficiency		RMSE^r.e.^	0.02	0.16	0.17	0.15	0.06	0.10	0.54
			${\hat{E}}^{\text{r.e.}}$	0.03	0.01	0.23	0.14	0.12	0.14	0.28
			${\hat{\sigma }}^{\text{r.e.}}$	0.09	0.02	0.02	0.03	0.04	0.03	0.04
	Mapping	≤57%	RMSE^m.a.^	0.11	0.17	0.23	0.17	0.10	0.11	0.16
	accuracy	≤8%	RMSE^m.a.^	0.17	0.22	0.32	0.45	0.28	0.26	0.40
$R_{i^{*},\text{QTL}}=0.18$	Relative efficiency		RMSE^r.e.^	0.00	0.18	0.46	0.21	0.14	0.14	0.40
			${\hat{E}}^{\text{r.e.}}$	0.00	0.18	0.39	0.35	0.31	0.34	0.31
			${\hat{\sigma }}^{\text{r.e.}}$	0.00	0.06	0.02	0.03	0.05	0.04	0.06
	Mapping	≤57%	RMSE^m.a.^	0.06	0.29	0.27	0.33	0.16	0.21	0.28
	accuracy	≤8%	RMSE^m.a.^	0.10	0.34	0.36	0.46	0.29	0.30	0.31
$R_{i^{*},\text{QTL}}=0.08$	Relative efficiency		RMSE^r.e.^	0.00	0.76	1.00	1.00	1.04	1.04	0.72
			${\hat{E}}^{\text{r.e.}}$	0.00	0.24	0.35	0.33	0.31	0.37	0.34
			${\hat{\sigma }}^{\text{r.e.}}$	0.00	0.06	0.04	0.05	0.06	0.05	0.08
	Mapping	≤57%	RMSE^m.a.^	0.13	0.66	0.58	0.54	0.51	0.58	0.55
	accuracy	≤8%	RMSE^m.a.^	0.18	0.71	0.66	0.71	0.62	0.69	0.59

▪$R_{i^{*},\text{QTL}}$: Multiallelic measure of LD between the simulated QTL and the haplotypes harboring it.

▪ RMSE^r.e.^: Root mean square error of $\theta_{\text{r.e.}}^{P}$ with respect to *θ*_*QTL*_ (cM).

▪${\hat{E}}^{\text{r.e.}}$: Expected value of the matrix distance at $\theta_{\text{r.e.}}^{P}$.

▪${\hat{\sigma }}^{\text{r.e.}}$: Standard error of the matrix distance at $\theta_{\text{r.e.}}^{P}$.

▪ RMSE^m.a.^: Root mean square error of $\theta_{\text{m.a.}}^{P}$ with respect to *θ*_*QTL*_ (cM).

In Table 1, IBS mQTL refers to the IBS _m_ predictor applied to the data set containing the causal variants. This situation was examined as a gold standard. As shown in Table 1 and as expected, IBS mQTL provided the best mapping accuracy since the data set used contained the causal variants and both the simulated QTL and the analyzed markers were biallelic. However, it should be noted that the RMSE ^m.a.^ for IBS mQTL was never equal to 0. This is principally due to the error term in the probabilistic models for hypothesis testing. RMSE ^r.e.^ for IBS mQTL was also not equal to 0 when LD was highest ($R_{i^{*},\text{QTL}}=0.52$). This was due to a nearby marker which was in complete LD with the SNP that simulated the QTL (i.e. the biallelic LD was complete). Consequently the argument of the minimum (argmin) for the set of matrix distances was not unique.

**Figure 5:**
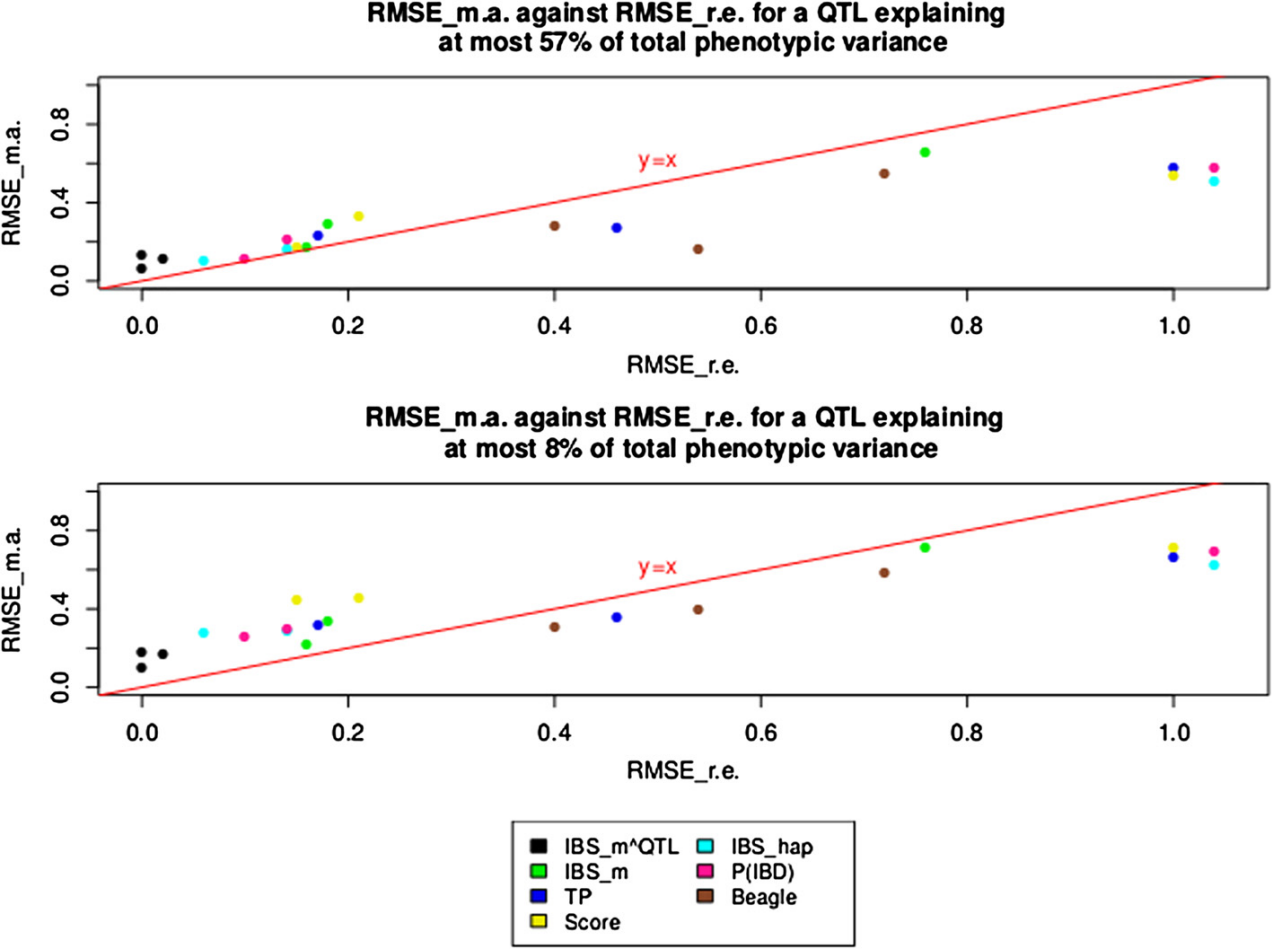
**RMSE**^**m.a. **^**against RMSE**^**r.e. **^**for the compared AIP according to different QTL effects across all LD levels.**

In Table 1 both RMSE ^r.e.^ and RMSE ^m.a.^ increased globally for all predictors when LD decreased in the vicinity of the QTL. RMSE ^r.e.^ and RMSE ^m.a.^ were highly correlated, regardless of the QTL effect. Across all LD levels, the Spearman correlation coefficient between these two quantities was equal to 0.89 (or 0.91) when the QTL effect explained at most 57% (or 8%) of the total variance, respectively (Figure 5).

**Figure 6:**
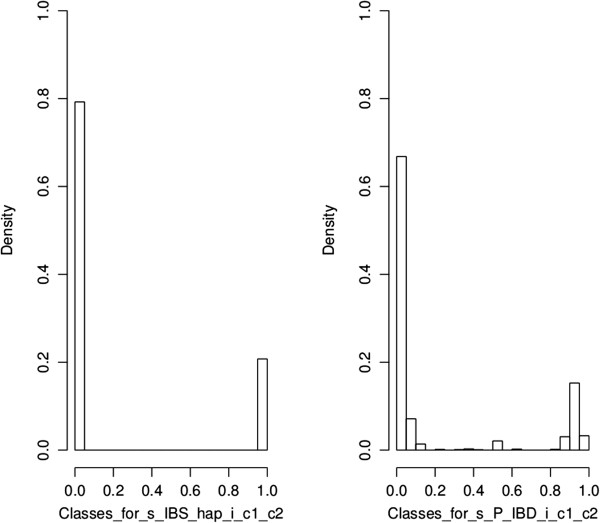
**Distribution of values for IBS**_**hap **_**and P(IBD) between chromosome segments around the simulated QTL for the moderate LD situation (), example for one simulation.** The class width for the IBD probabilities is equal to 0.05.

Each dot in Figure 5 represents RMSE ^m.a.^ against RMSE ^r.e.^ for one of the AIP at a particular LD level. In Table 1, the IBS _hap_ predictor was often the most accurate and efficient AIP when the data was analyzed without the QTL. However, the P(IBD) predictor showed similar mapping and efficiency results to IBS _hap_. As defined by [1], the P(IBD) predictor relies on the IBS state of alleles between haplotype markers which suggests that IBS _hap_ and P(IBD) may perform similarly in some conditions [41]. Indeed, the distribution of IBD probabilities in the vicinity of a simulated QTL was almost bimodal (0 or 1) among the different pairs of chromosome segments for the different sets of simulations, and thus similar to the distribution of the values for IBS _hap_ between the segments. To illustrate this phenomenon, Figure 6 provides an example of distributions for the values of P(IBD) and IBS _hap_, for one gene-drop simulation, between pairs of chromosome segments around the simulated QTL for the moderate LD situation ($R_{i^{*},\text{QTL}}=0.18$).

**Figure 7:**
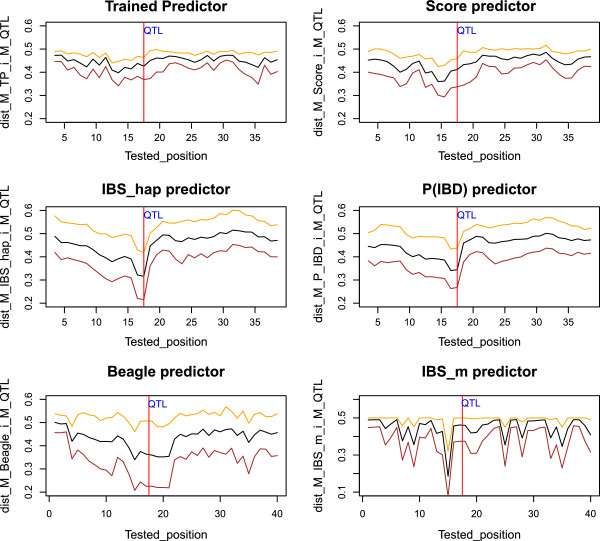
**The mean and sample quantiles at 2.5% and 97.5% of **$d_{1}({\text{M}}^{P,i},{\text{M}}^{\text{QTL}})$** (black, brown and orange respectively) for TP, Score, IBS**_***hap***_**, P(IBD), Beagle and IBS**_***m***_**, with respect to the tested position and the simulated QTL position (red line) for the moderate LD situation (**$R_{i^{*},\text{QTL}}=0.18$**).**

IBS _hap_ and P(IBD) also showed similar patterns at a set of tested positions for the matrix distances $d_{1}({\text{M}}^{P,i},{\text{M}}^{\text{QTL}})$. Figure 7 shows an example for the mean and the sample quantiles at 2.5 and 97.5% for $d_{1}({\text{M}}^{P,i},{\text{M}}^{\text{QTL}})$ at each tested position for the six AIP, from 200 gene-drop simulations with a QTL simulated for the moderate LD situation ($R_{i^{*},\text{QTL}}=0.18$).

As observed in Figure 7, the minima of the curves for the mean and the sample quantiles at 2.5 and 97.5% of the matrix distance distributions almost coincide with the QTL position for IBS _hap_ and P(IBD). For these two predictors, the three curves also show a smooth decreasing behavior as the tested position gets closer to the simulated QTL. This behavior shows the ability of IBS _hap_ and P(IBD) to capture LD structure along the chromosomes with respect to the simulated QTL, for different gene-drop simulations. It is interesting to note that IBS _hap_ and P(IBD) show similar patterns for the mean and the sample quantiles curves. However, the minimum of each of the three curves in Figure 7 is lower for IBS _hap_ than for P(IBD). Note that the patterns of the matrix distances for IBS _hap_ in Figure 7 are explained by equation (4) and Figure 2. That is, the matrix distance will decrease for IBS _hap_ due to the expected increase of the multiallelic LD, as the tested position moves toward the QTL position. In the same way, the patterns of the matrix distances for P(IBD) in Figure 7 are explained according to Figures 2 and 6. That is, P(IBD) will behave slightly differently from IBS _hap_, according to Figures 2 and 6, when taking equations (3) and (5) into account.

As shown in Figure 7, the other predictors cannot capture the LD structure along the chromosomes with respect to the simulated QTL as well as IBS _hap_ and P(IBD); this is particularly the case for Score and even more for TP. For the latter two predictors, $d_{1}({\text{M}}^{P,i},{\text{M}}^{\text{QTL}})$ shows little variability and is low on average across the tested positions. This could explain the lack of a clear ranking between the mapping accuracies of TP and Score in Table 1. For Beagle, a good relative efficiency and mapping accuracy was observed for the lowest LD situation ($R_{i^{*},\text{QTL}}=0.08$) in Table 1, compared to all the other predictors, when the QTL effect was low. Note that AIP that are based on haplotypes and that do not perform haplotype clustering like Beagle, may not be at an advantage for a low LD situation. For example, the matrix distance for IBS _hap_, as defined by equation (4), will not decrease if there is little LD between local haplotypes and QTL alleles. Therefore, haplotype clustering is necessary for such situations. Moreover, these AIP will intrinsically provide an excess of degrees of freedom for testing association if the QTL is biallelic, while not compensating for the low LD captured in the matrix distance. Hence, AIP based on haplotype clustering can provide higher mapping accuracy for low LD situations.

## Discussion

### Matrix distance properties

The present study showed that the QTL mapping accuracy of AIP is highly correlated to the tested position that minimizes the matrix distance defined for comparison. The use of the matrix distance to compare various AIP has many advantages for methodology development and validation. First, it is independent of phenotype simulation processes and statistical tests that are commonly used to compare QTL mapping accuracy of different AIP [4,8,23,25]. Indeed the phenotype simulation process, when based on certain specific assumptions, may favor some AIP over others: for example, IBD-based AIP might be at an advantage if the phenotypes are simulated only according to population history. The statistical test used may also favor some AIP, such as IBS _hap_, IBS _m_ and Beagle, over others due to numerical stability when estimating variance components. As such, solving mixed model equations when covariance matrices are close to singularity due to AIP computation has been reported as an issue, and clustering strategies for haplotypes, which actually modify the properties of the AIP matrices, have been proposed to facilitate computation [42,43]. The major drawback of the matrix distance approach is related to this advantage: a particularly efficient AIP or a particularly efficient haplotype size, identified from the matrix distance, that can not be used in association studies would be of no value. Another advantage of the matrix distance approach is that computation time is highly reduced compared to association studies, so numerous comparisons can be done. In the present study, the relative efficiency of the AIP was consistent with the results for QTL mapping accuracy, regardless of the QTL effects and LD patterns. Therefore, the concept of relative efficiency was proven useful to compare AIP and avoid time-consuming association studies on simulated data. Combining the relative efficiency with the mapping accuracy of predictors could also be helpful to gain a better understanding of the underlying mechanisms in an association study.

### Comparing AIP

The results showed that the most accurate AIP for mapping were those that best captured LD between a tested position and a QTL. This was proposed from algebraic developments in the simplest situations and validated using real data and simulations. The matrix distance can be written for any AIP as a sum of functions of LD coefficients, and more precisely for the IBS _hap_ predictor as a sum of concave polynomials of LD coefficients. When LD was moderate to high around the QTL, the IBS _hap_ predictor was the most efficient and accurate matrix for mapping. For a biallelic QTL, the domains of values for which some of these concave polynomials can either decrease or increase with increasing LD was shown in our developments as limited to extreme allele frequencies for the haplotypes and QTL. Additionally, continuous AIP in [0,1] were shown to deteriorate the matrix distance generally when LD between a tested position and the QTL increased. This was observed on two unrelated data sets, which showed that this behavior is not related to the marker density or population history. All LD measures are based on counting occurrences for discrete events at distinct loci to quantify non-random association [37,38], which thus explains the algebraic and simulation results for discrete and continuous AIP when a relatively high LD is available for detection. The pig example was built using 235 haplotypes and 25 gene-drop generations, a realistic situation with regard to the effective population size. However, the impact of the resulting long-range haplotypic identity, which depends strongly on the population size and mating strategies, on the relative values of the considered AIP should be investigated.

Despite using two contrasting data sets in terms of marker density and population history, P(IBD) always behaved very similarly to IBS _hap_. When extending the calculations to longer haplotypes (results not shown), a similar behavior was observed. Yet advantages have been reported for P(IBD) compared to IBS _hap_ in some situations. For example, Roldan *et al.*[43] showed better accuracy for P(IBD) compared to IBS _hap_, after a clustering step for haplotypes when marker intervals were equal to 0.05 cM between SNPs, but not when they reached 0.25 cM. However in Roldan *et al.*[43], different statistical models were applied to P(IBD) versus IBS _hap_ (mixed model *versus* fixed effects model respectively). Hence, these two AIP were not compared on the same basis. For instance, Boleckova *et al.*[44] showed that statistical models in which haplotypes were fitted as random effects performed better than those in which they were fitted as fixed effects. When both LD and the QTL effect were low, Beagle showed a relatively good efficiency and mapping accuracy. It was not possible to derive algebraical comparisons between AIP when LD was low, but this, together with earlier studies that point out that continuous advanced methods are more efficient than simple IBS _hap_, suggests that some continuous AIP in [0,1] may provide efficiency when LD between markers and a QTL is reduced.

### Extending the results to multiallelic QTL

In the present study, we considered a biallelic QTL for algebra and simulations. Yet the algebraic derivation of the matrix distance can be generalized to a multiallelic QTL without difficulty [see Additional file 1]. As suggested by these developments, for a multiallelic QTL, the relationship between continuous predictions of allelic identity at a tested position and the corresponding LD coefficients will tend to be looser than for discrete predictions. In addition, the matrix distance for the IBS _hap_ predictor can always be written as a sum of concave polynomials of LD coefficients for any degree of allelism at the QTL.

## Conclusion

The IBS _hap_ predictor can always capture multiallelic LD between a tested position and a QTL, regardless of the degree of allelism at the QTL. The IBS _hap_ predictor also has the advantage of being simple, fast and numerically stable when used in association studies. Therefore, it is suggested that, for studies with a high density of markers and for which LD between markers and the causal variants is likely to be high, the use of the IBS _hap_ predictor is recommended.

## Competing interests

The authors declare that they have no competing interests.

## Authors’ contributions

LJ derived the analytical results, performed the simulations and wrote the manuscript. LJ, JME and HG were involved in the conception of the study. All authors read and approved the final manuscript.

## Supplementary Material

Additional file 1**Algebraic derivations of formulas in the main text.** This file contains all the algebraic derivations for expressions (1) to (6) and the generalization of the matrix distance, as a sum of concave functions of LD coefficients when  IBS _hap_, for the case of a multiallelic QTL.Click here for file

Additional file 2 **Domains of LD coefficients and boundary conditions for the critical values of each*Q***_***p***_** function.** This file contains the domain of values for the multiallelic LD coefficients, the boundary conditions for the critical value of each *Q*_*p*_ function in expression (4) and the relation between the sum of the squared deviations and $D_{i,\text{QTL}}^{2}$. Click here for file

## References

[B1] Meuwissen THE Goddard ME Prediction of identity by descent probabilities from marker haplotypes Genet Sel Evol 2001 33 605 634 10.1186/1297-9686-33-6-605 11742632 PMC2705394

[B2] Li J Jiang T Haplotype-based linkage disequilibrium mapping via direct data mining Bioinformatics 2005 21 4384 4393 10.1093/bioinformatics/bti732 16249262

[B3] Browning SR Multilocus association mapping using variable-length Markov chains Am J Hum Genet 2006 78 903 913 10.1086/503876 16685642 PMC1474089

[B4] Pong-Wong R George AW Woolliams JA Haley CS A simple and rapid method for calculating identity-by-descent matrices using multiple markers Genet Sel Evol 2001 33 453 471 10.1186/1297-9686-33-5-453 11712969 PMC2705399

[B5] Bercovici S Meek C Wexler Y Geiger D Estimating genome-wide IBD sharing from SNP data via an efficient hidden Markov model of LD with application to gene mapping Bioinformatics 2010 26 i175 i182 10.1093/bioinformatics/btq204 20529903 PMC2881389

[B6] Druet T Georges M A hidden Markov model combining linkage and linkage disequilibrium information for haplotype reconstruction and quantitative trait locus fine mapping Genetics 2010 184 789 798 10.1534/genetics.109.108431 20008575 PMC2845346

[B7] Akey J Jin L Xiong M: Haplotypes vs single marker linkage disequilibrium tests: what do we gain? Eur J Hum Genet 2001 9 291 300 10.1038/sj.ejhg.5200619 11313774

[B8] Abdallah J Goffinet B Cierco-Ayrolles C Pérez-Enciso M Linkage disequilibrium fine mapping of quantitative trait loci: a simulation study Genet Sel Evol 2003 35 513 532 10.1186/1297-9686-35-6-513 12939203 PMC2697979

[B9] Cardon LR Abecasis GR Using haplotype blocks to map human complex trait loci Trends Genet 2003 19 136 140 10.1016/S0168-9525(03)00022-2 12615007

[B10] Clark AG The role of haplotypes in candidate gene studies Genet Epidemiol 2004 27 321 333 10.1002/gepi.20025 15368617

[B11] Schaid DJ Evaluating associations of haplotypes with traits Genet Epidemiol 2004 27 348 364 10.1002/gepi.20037 15543638

[B12] Browning BL Browning SR Efficient multilocus association testing for whole genome association studies using localized haplotype clustering Genet Epidemiol 2007 31 365 375 10.1002/gepi.20216 17326099

[B13] Chen Y Li X Li J A novel approach for haplotype-based association analyzis using family data BMC Bioinformatics 2010 11 S45 10.1186/1471-2105-11-S1-S45 20122219 PMC3009518

[B14] Lin WY Yi N Zhi D Zhang K Gao G Tiwari HK Liu N Haplotype-based methods for detecting uncommon causal variants with common SNPs Genet Epidemiol 2012 36 572 582 10.1002/gepi.21650 22706849 PMC3513398

[B15] Knüppel S Esparza-Gordillo J Marenholz I Holzhütter HG Bauerfeind A Ruether A Weidinger S Lee YA Rohde K Multi-locus stepwise regression: a haplotype based algorithm for finding genetic associations applied to atopic dermatitis BMC Med Genet 2012 13 8 22284537 10.1186/1471-2350-13-8 PMC3398269

[B16] Li M Wing HW Art BO A sparse transmission disequilibrium test for haplotypes based on Bradley-Terry graphs Hum Hered 2012 73 52 61 10.1159/000335937 22398955 PMC3357149

[B17] Risch N Merikangas K The future of genetic studies of complex human diseases Science 1996 273 1516 1517 10.1126/science.273.5281.1516 8801636

[B18] Terwilliger JD Weiss KM Linkage disequilibrium mapping of complex disease: fantasy or reality? Curr Opin Biotechnol 1998 9 578 594 10.1016/S0958-1669(98)80135-3 9889136

[B19] Jorde LB Linkage disequilibrium and the search for complex disease genes Genome Res 2000 10 1435 1444 10.1101/gr.144500 11042143

[B20] Weiss KM Clark AG Linkage disequilibrium and the mapping of complex human traits Trends in Genet 2002 18 19 24 10.1016/S0168-9525(01)02550-1 11750696

[B21] Slatkin M Disequilibrium mapping of a quantitative-trait locus in an expanding population Am J Hum Genet 1999 64 1764 1772 10330364 10.1086/302413 PMC1377920

[B22] Farnir F Coppieters W Arranz JJ Berzi P Cambisano N Grisart B Karim L Marcq F Moreau L Mni M Nezer C Simon P Vanmanshoven P Wagenaar D George M Extensive genome-wide linkage disequilibrium in cattle Genome Res 2000 10 220 227 10.1101/gr.10.2.220 10673279

[B23] Meuwissen THE Goddard ME Fine mapping of quantitative trait loci using linkage disequilibria with closely linked marker loci Genetics 2000 155 421 430 10790414 10.1093/genetics/155.1.421 PMC1461086

[B24] Remington DL Thornsberry JM Matsuoka Y Wilson LM Whitt SR Doebley J Kresovich S Goodman MM Buckler ES Structure of linkage disequilibrium and phenotypic associations in the maize genome Proc Natl Acad Sci USA 2001 98 11479 11484 10.1073/pnas.201394398 11562485 PMC58755

[B25] He W Fernando RL Dekkers JCM Gilbert H A gene frequency model for QTL mapping using Bayesian inference Genet Sel Evol 2010 42 21 10.1186/1297-9686-42-21 20540762 PMC2901203

[B26] Grapes L Dekkers JCM Rothschild MF Fernando RL Comparing linkage disequilibrium-based methods for fine mapping quantitative trait loci Genetics 2004 166 1561 1570 10.1534/genetics.166.3.1561 15082569 PMC1470790

[B27] Browning BL Browning SR Haplotypic analysis of Wellcome Trust Case Control Consortium data Human Genet 2008 123 273 280 10.1007/s00439-008-0472-1 18224336 PMC2384233

[B28] Henderson CR Best linear unbiased estimation and prediction under a selection model Biometrics 1975 31 423 447 10.2307/2529430 1174616

[B29] Henderson CR A simple method for computing the inverse of a numerator relationship matrix used in prediction of breeding values Biometrics 1976 32 69 83 10.2307/2529339

[B30] Dempster AP Laird NM Rubin DB Maximum likelihood from in-complete data via the EM algorithm Roy Statist Soc Ser B 1977 39 1 38

[B31] Patterson HD Thompson R Recovery of inter-block information when block sizes are unequal Biometrika 1971 58 545 554 10.1093/biomet/58.3.545

[B32] Harville DA Bayesian inference for variance components using only error contrasts Biometrika 1974 61 383 385 10.1093/biomet/61.2.383

[B33] Foulley JL EM algorithm: theory and application to the mixed model J Soc Fr Stat 2002 143 57 109

[B34] Calus MPL Meuwissen THE Windig JJ Knol EF Schrooten C Vereijken ALJ Veerkamp RF Effects of the number of markers per haplotype and clustering of haplotypes on the accuracy of QTL mapping and prediction of genomic breeding values Genet Sel Evol 2009 41 11 10.1186/1297-9686-41-11 19284677 PMC3225874

[B35] Grapes L Firat MZ Dekkers JCM Rothschild MF Fernando RL Optimal haplotype structure for linkage disequilibrium-based fine mapping of quantitative trait loci using identity by descent Genetics 2005 172 1955 1965 10.1534/genetics.105.048686 16322505 PMC1456285

[B36] Ramos AM Crooijmans RP Affara NA Amaral AJ Archibald AL Beever JE Bendixen C Churcher C Clark R Dehais P Hansen MS Hedegaard J Hu ZL Kerstens HH Law AS Megens HJ Milan D Nonneman DJ Rohrer GA Rothschild MF Smith TP Schnabel RD Van Tassell CP Taylor JF Wiedmann RT Schook LB Groenen MA Design of a high density SNP genotyping assay in the pig using SNPs identified and characterized by next generation sequencing technology PLoS ONE 2009 4 e6524 10.1371/journal.pone.0006524 19654876 PMC2716536

[B37] Hedrick PW Thomson G A two-locus neutrality test: applications to humans, E. coli and Lodgepole pine Genetics 1985 112 135 156 3510942 10.1093/genetics/112.1.135 PMC1202687

[B38] Hedrick PW Gametic disequilibrium measures: proceed with caution Genetics 1987 117 331 341 3666445 10.1093/genetics/117.2.331 PMC1203208

[B39] Maurer HP Knaak C Melchinger AE Ouzunova M Frisch M Linkage disequilibrium between SSR markers in six pools of elite lines of an european breeding program for hybrid maize Maydica 2006 51 269 279

[B40] Ytournel F Teyssèdre S Roldan D Erbe M Simianer H Boichard D Gilbert H Druet T Legarra A LDSO: A program to simulate pedigrees and molecular information under various evolutionary forces J Anim Breed Genet 2012 129 417 421 10.1111/j.1439-0388.2011.00986.x 22963363

[B41] Ytournel F Gilbert H Boichard D Concordance between IBD probabilities and linkage disequilibrium Proceedings of European Federation of Animal Science Annual Meeting; 26 August 2007; Dublin 2007 1248 1248 [http://www.eaap.org/Previous_Annual_Meetings/2007Dublin/Papers/S38_1248_Ytournel.pdf]

[B42] Druet T Fritz S Boussaha M Ben-Jemaa S Guillaume F Derbala D Zelenika D Lechner D Charon C Boichard D Gut I Eggen A Gautier M Fine mapping of quantitative trait loci affecting female fertility in dairy cattle on BTA03 using a dense single-nucleotide polymorphism map Genetics 2008 178 2227 2235 10.1534/genetics.107.085035 18430945 PMC2323811

[B43] Roldan DL Gilbert H Henshall JM Legarra A Elsen JM Fine-mapping quantitative trait loci with a medium density marker panel: efficiency of population structures and comparison of linkage disequilibrium linkage analysis models Genet Res Camb 2012 94 223 234 10.1017/S0016672312000407 22950902 PMC3487687

[B44] Boleckova J Christensen OF Sørensen P Sahana G Strategies for haplotype-based association mapping in a complex pedigreed population Czech J Anim Sci 2012 1 1 9

