# Supplementary material for: Using haplotypes for the prediction of allelic identity to fine-map QTL: characterization and properties
Source: Genet Sel Evol. 2014 Jul 14;46(1):45. doi: 10.1186/1297-9686-46-45 (PMC4223544; doi:10.1186/1297-9686-46-45)
Supplement: Additional file 1 — Algebraic derivations of formulas in the main text. This file contains all the algebraic derivations for expressions (1) to (6) and the generalization of the matrix distance, as a sum of concave functions of LD coefficients when IBS hap, for the case of a multiallelic QTL. [file 1297-9686-46-45-S1.pdf]

Additional file 1

Details of the algebraic derivations of the formulas in the main text

Algebraic derivation of expression (1)

Let $K = 2^t$ be the number of possible haplotypes, at locus i, for a sliding window of t markers. Let $\mathbb{1}_{\{u_{QTL,c_1,c_2}=1\}}$ and $\mathbb{1}_{\{u_{QTL,c_1,c_2}=0\}}$ be the following indicator functions:

$$\mathbb{1}_{\{u_{QTL,c_1,c_2}=1\}} = \begin{cases} 1 \text{ if } (c_1, c_2) \text{ have identical alleles at the QTL} \\ 0 \text{ else} \end{cases}$$

$$\mathbb{1}_{\{u_{QTL,c_1,c_2}=0\}} = \begin{cases} 1 \text{ if } (c_1, c_2) \text{ have non-identical alleles at the QTL} \\ 0 \text{ else} \end{cases}$$

We have:

$$\begin{aligned} d_1(\mathbf{M}^{\mathcal{P},i}, \mathbf{M}^{QTL}) &= \frac{1}{4n^2} \sum_{c_1=1}^{2n} \sum_{c_2=1}^{2n} |s^{\mathcal{P}}_{i,c_1,c_2} - u_{QTL,c_1,c_2}| \\ &= \frac{1}{4n^2} \sum_{c_1=1}^{2n} \sum_{c_2=1}^{2n} \mathbb{1}_{\{u_{QTL,c_1,c_2}=1\}} |s^{\mathcal{P}}_{i,c_1,c_2} - 1| + \frac{1}{4n^2} \sum_{c_1=1}^{2n} \sum_{c_2=1}^{2n} \mathbb{1}_{\{u_{QTL,c_1,c_2}=0\}} |s^{\mathcal{P}}_{i,c_1,c_2} - 0| \end{aligned}$$

Let E_{h_p} be the set of chromosome segments carrying haplotype h_p ($p \in \{1, .., K\}$) at locus i. We have:

$$\begin{aligned} d_1(\mathbf{M}^{\mathcal{P},i}, \mathbf{M}^{QTL}) =& \frac{1}{4n^2} \sum_{c_1=1}^{2n} \sum_{c_2=1}^{2n} \sum_{p=1}^{K} \mathbb{1}_{\{c_1 \in E_{h_p}\}} \sum_{q=1}^{K} \mathbb{1}_{\{c_2 \in E_{h_q}\}} \mathbb{1}_{\{u_{QTL,c_1,c_2}=1\}} |s^{\mathcal{P}}_{i,c_1,c_2} - 1| \\ &+ \frac{1}{4n^2} \sum_{c_1=1}^{2n} \sum_{c_2=1}^{2n} \sum_{p=1}^{K} \mathbb{1}_{\{c_1 \in E_{h_p}\}} \sum_{q=1}^{K} \mathbb{1}_{\{c_2 \in E_{h_q}\}} \mathbb{1}_{\{u_{QTL,c_1,c_2}=0\}} |s^{\mathcal{P}}_{i,c_1,c_2} - 0| \end{aligned}$$

where $\mathbb{1}_{\{c_1 \in E_{h_p}\}}$ and $\mathbb{1}_{\{c_2 \in E_{h_q}\}}$ are the indicator functions of the events $\{c_1 \in E_{h_p}\}$ and $\{c_2 \in E_{h_q}\}$ respectively. Indeed $\sum_{p=1}^{K} \mathbb{1}_{\{c_1 \in E_{h_p}\}}$ and $\sum_{q=1}^{K} \mathbb{1}_{\{c_2 \in E_{h_q}\}}$ are always equal to one. $d_1(\mathbf{M}^{\mathcal{P},i}, \mathbf{M}^{QTL})$ can thus be expressed as:

$$
\begin{aligned}
d_1(\mathbf{M}^{\mathcal{P},i}, \mathbf{M}^{QTL}) = & \sum_{p=1}^{K}\sum_{q=1}^{K}\frac{1}{4n^2}\sum_{c_1=1}^{2n}\sum_{c_2=1}^{2n}\mathbb{1}_{\left\{u_{QTL,c_1 \in E_{h_p},c_2 \in E_{h_q}}=1\right\}}|s^{\mathcal{P}}_{i,h_p,h_q} - 1| \\
& + \sum_{p=1}^{K}\sum_{q=1}^{K}\frac{1}{4n^2}\sum_{c_1=1}^{2n}\sum_{c_2=1}^{2n}\mathbb{1}_{\left\{u_{QTL,c_1 \in E_{h_p},c_2 \in E_{h_q}}=0\right\}}|s^{\mathcal{P}}_{i,h_p,h_q} - 0|
\end{aligned}
$$

where $\frac{1}{4n^2}\sum_{c_1=1}^{2n}\sum_{c_2=1}^{2n}\mathbb{1}_{\left\{u_{QTL,c_1 \in E_{h_p},c_2 \in E_{h_q}}=1\right\}} = f(c_1 \in E_{h_p}, c_2 \in E_{h_q}, \text{identical alleles at the QTL})$ is the frequency of chromosome segments, at locus i, carrying h_p and h_q and having identical alleles at the QTL. Since $\{c_1 \in E_{h_p}\}$ and $\{c_2 \in E_{h_q}\}$ are independent events we have:

$$
f(c_1 \in E_{h_p}, c_2 \in E_{h_q}, \text{identical alleles at the QTL}) = p^{QTL}_{i,h_p,h_q} f_{i,h_p} f_{i,h_q}
$$

where p^{QTL}_{i,h_p,h_q} is the proportion of identical alleles shared at the QTL by the couples of chromosomes carrying h_p and h_q at position i. f_{i,h_p} and f_{i,h_q} are the frequencies of haplotypes h_p and h_q at position i respectively.

Similarly we have:

$$
\begin{aligned}
\frac{1}{4n^2}\sum_{c_1=1}^{2n}\sum_{c_2=1}^{2n}\mathbb{1}_{\left\{u_{QTL,c_1 \in E_{h_p},c_2 \in E_{h_q}}=0\right\}} = & f(c_1 \in E_{h_p}, c_2 \in E_{h_q}, \text{non-identical alleles at the QTL}) \\
= & (1 - p^{QTL}_{i,h_p,h_q}) f_{i,h_p} f_{i,h_q}
\end{aligned}
$$

Consequently $d_1(\mathbf{M}^{\mathcal{P},i}, \mathbf{M}^{QTL})$ can be written as (1), i.e.

$$d_1(\mathbf{M}^{\mathcal{P},i}, \mathbf{M}^{QTL}) = \sum_{p=1}^{K}\sum_{q=1}^{K} f_{i,h_p} f_{i,h_q} \left[p^{QTL}_{i,h_p,h_q}(1 - s^{\mathcal{P}}_{i,h_p,h_q}) + (1 - p^{QTL}_{i,h_p,h_q}) s^{\mathcal{P}}_{i,h_p,h_q}\right] \quad (1)$$

Algebraic derivation of expression (2)

Let $(n_{h_p})_{1 \le p \le K}$ be the counts of the possible haplotypes at a tested position i. And let $(n_{h_p a_l})_{\substack{1 \le p \le K \\ 1 \le l \le 2}}$ be the counts of the $2K$ possible haplotypes defined between i and a QTL. Expression (1) can be re-written as:

$$\begin{aligned} d_1(\mathbf{M}^{\mathcal{P},i}, \mathbf{M}^{QTL}) &= \sum_{p=1}^{K}\sum_{q=1}^{K} \frac{n_{h_p}}{2n}\frac{n_{h_q}}{2n}\left[\frac{[n_{h_p a_1} n_{h_q a_1} + n_{h_p a_2} n_{h_q a_2}]}{n_{h_p} n_{h_q}}(1 - s^{\mathcal{P}}_{i,h_p,h_q}) \right. \\ &\quad \left. + \left(\frac{n_{h_p} n_{h_q} - [n_{h_p a_1} n_{h_q a_1} + n_{h_p a_2} n_{h_q a_2}]}{n_{h_p} n_{h_q}}\right) s^{\mathcal{P}}_{i,h_p,h_q}\right] \\ &= \sum_{p=1}^{K}\sum_{q=1}^{K} \left[\left[f^{QTL}_{i,h_p a_1} f^{QTL}_{i,h_q a_1} + f^{QTL}_{i,h_p a_2} f^{QTL}_{i,h_q a_2}\right](1 - s^{\mathcal{P}}_{i,h_p,h_q}) \right. \\ &\quad \left. + \left(\frac{(n_{h_p a_1} + n_{h_p a_2})(n_{h_q a_1} + n_{h_q a_2})}{4n^2} - f^{QTL}_{i,h_p a_1} f^{QTL}_{i,h_q a_1} - f^{QTL}_{i,h_p a_2} f^{QTL}_{i,h_q a_2}\right) s^{\mathcal{P}}_{i,h_p,h_q}\right] \end{aligned}$$

which simplifies to (2):

$$\begin{aligned} d_1(\mathbf{M}^{\mathcal{P},i}, \mathbf{M}^{QTL}) = \sum_{p=1}^{K}\sum_{q=1}^{K} &\left[\left[f^{QTL}_{i,h_p a_1} f^{QTL}_{i,h_q a_1} + f^{QTL}_{i,h_p a_2} f^{QTL}_{i,h_q a_2}\right](1 - s^{\mathcal{P}}_{i,h_p,h_q}) \right. \\ &\left. + \left[f^{QTL}_{i,h_p a_2} f^{QTL}_{i,h_q a_1} + f^{QTL}_{i,h_p a_1} f^{QTL}_{i,h_q a_2}\right] s^{\mathcal{P}}_{i,h_p,h_q}\right] \quad (2) \end{aligned}$$

Algebraic derivation of expression (3)

Let $f^{QTL}_{i,h_p a_1} = f_{i,h_p} f_{a_1} + \mathbf{\Delta_p} = \alpha_p + \mathbf{\Delta_p}$ and $f^{QTL}_{i,h_p a_2} = f_{i,h_p} f_{a_2} - \mathbf{\Delta_p} = \tilde{\alpha}_p - \mathbf{\Delta_p}$. Note that we have $\sum_{p=1}^{K} \mathbf{\Delta_p} = \sum_{p=1}^{K} (f^{QTL}_{i,h_p a_1} - f_{i,h_p} f_{a_1}) = f_{a_1} - f_{a_1} \sum_{p=1}^{K} f_{i,h_p} = 0$. Replacing the haplotype frequencies in

expression (2) with the expressions including the α_p, $\tilde{\alpha}_p$ and $\mathbf{\Delta_p}$ terms (same for the frequencies depending on the $\alpha_q, \tilde{\alpha}_q$ and $\mathbf{\Delta_q}$ terms) gives:

$$
\begin{aligned}
d(\mathbf{M}^{\mathcal{P},i}, \mathbf{M}^{QTL}) = \sum_{p=1}^{k} \Big[& -4s^{\mathcal{P}}_{i,h_p,h_p}\mathbf{\Delta_p}^2 + \Big[\alpha_p - \tilde{\alpha}_p + 4s^{\mathcal{P}}_{i,h_p,h_p}(\tilde{\alpha}_p - \alpha_p) + \sum_{q \neq p}^{k}(\alpha_q - \tilde{\alpha}_q)\Big]\mathbf{\Delta_p} \\
& + \alpha_p^2 + \tilde{\alpha}_p^2 + s^{\mathcal{P}}_{i,h_p,h_p}(-\alpha_p^2 - \tilde{\alpha}_p^2 + 2\alpha_p\tilde{\alpha}_p) + \sum_{q \neq p}^{k} \alpha_p\alpha_q + \tilde{\alpha}_p\tilde{\alpha}_q \\
& + \sum_{q \neq p}^{k} s^{\mathcal{P}}_{i,h_p,h_q}\Big(-4\mathbf{\Delta_p}\mathbf{\Delta_q} + 2(\tilde{\alpha}_p - \alpha_p)\mathbf{\Delta_q} + 2(\tilde{\alpha}_q - \alpha_q)\mathbf{\Delta_p} \\
& - \alpha_p\alpha_q - \tilde{\alpha}_p\tilde{\alpha}_q + \tilde{\alpha}_p\alpha_q + \alpha_p\tilde{\alpha}_q\Big) \Big] \quad (*)
\end{aligned}
$$

Replacing $\mathbf{\Delta_q}$ with $-\mathbf{\Delta_p} - \sum_{l \neq p,q}^{K} \mathbf{\Delta_l}$ $\Big($ since $\sum_{p=1}^{k} \mathbf{\Delta_p} = 0 \Big)$ in (*) finally gives:

$$
\begin{aligned}
d_1(\mathbf{M}^{\mathcal{P},i}, \mathbf{M}^{QTL}) = & \sum_{p=1}^{K} \Big[\; 4\Big(\sum_{q \neq p}^{K} s^{\mathcal{P}}_{i,h_p,h_q} - s^{\mathcal{P}}_{i,h_p,h_p}\Big)\mathbf{\Delta_p^2} + \Big[\; \alpha_p - \tilde{\alpha}_p + 4s^{\mathcal{P}}_{i,h_p,h_p}(\tilde{\alpha}_p - \alpha_p) \\
& + \sum_{q \neq p}^{K} \Big(\alpha_q - \tilde{\alpha}_q + s^{\mathcal{P}}_{i,h_p,h_q}\big(4\sum_{l \neq p,q}^{K} \mathbf{\Delta_l} + 2(\alpha_p - \tilde{\alpha}_p) + 2(\tilde{\alpha}_q - \alpha_q)\;\big)\;\Big)\;\Big]\mathbf{\Delta_p} \\
& + \alpha_p^2 + \tilde{\alpha}_p^2 + s^{\mathcal{P}}_{i,h_p,h_p}(-\alpha_p^2 - \tilde{\alpha}_p^2 + 2\alpha_p\tilde{\alpha}_p) + \sum_{q \neq p}^{K} \alpha_p\alpha_q + \tilde{\alpha}_p\tilde{\alpha}_q \\
& + \sum_{q \neq p}^{K} s^{\mathcal{P}}_{i,h_p,h_q}\Big(2(\alpha_p - \tilde{\alpha}_p)\sum_{l \neq p,q}^{K} \mathbf{\Delta_l} - \alpha_p\alpha_q - \tilde{\alpha}_p\tilde{\alpha}_q + \tilde{\alpha}_p\alpha_q + \alpha_p\tilde{\alpha}_q\Big) \; \Big]
\end{aligned}
$$

Hence $d_1(\mathbf{M}^{\mathcal{P},i}, \mathbf{M}^{QTL})$ can be expressed as (3):

$$
\begin{aligned}
d_1(\mathbf{M}^{\mathcal{P},i}, \mathbf{M}^{QTL}) = & \sum_{p=1}^{K} \Big[\; 4\Big(\sum_{q \neq p}^{K} s^{\mathcal{P}}_{i,h_p,h_q} - s^{\mathcal{P}}_{i,h_p,h_p}\Big)\mathbf{\Delta_p^2} + \Psi^{\mathcal{P}}_{pq}(\Delta_{l \neq p,q})\mathbf{\Delta_p} + \Phi^{\mathcal{P}}_{pq}(\Delta_{l \neq p,q}) \; \Big] \\
= & \xi^{\mathcal{P}}(\mathbf{\Delta_1}, .., \mathbf{\Delta_K}) \qquad (3)
\end{aligned}
$$

Algebraic derivation of expression (4)

Expression (4) is obtained directly from expression (3) for $s^{\mathcal{P}}_{i,h_p,h_p} = 1$ and $s^{\mathcal{P}}_{i,h_p,h_q} = 0$ when $\mathcal{P} = \text{IBS}_{\text{hap}}$.

Algebraic derivation of expressions (5) and (6)

Considering that only two haplotypes exist among the K possible ones is the same as setting $K = 2$. Expression (5) can be obtained directly by reducing expression (4) for the case where $K = 2$. However another derivation is given here so as to exhibit other properties, such as a lower bound, for the matrix distance. For $K = 2$ expression (2) becomes:

$$
\begin{aligned}
d_1(\mathbf{M}^{\mathcal{P},i}, \mathbf{M}^{QTL}) =& ({f^{QTL}_{i,h_1a_1}}^2 + {f^{QTL}_{i,h_1a_2}}^2) - s^{\mathcal{P}}_{i,h_1,h_1}(f^{QTL}_{i,h_1a_1} - f^{QTL}_{i,h_1a_2})^2 \\
&+ 2(f^{QTL}_{i,h_1a_1} f^{QTL}_{i,h_2a_1} + f^{QTL}_{i,h_1a_2} f^{QTL}_{i,h_2a_2})(1 - s^{\mathcal{P}}_{i,h_1,h_2}) \\
&+ 2(f^{QTL}_{i,h_1a_2} f^{QTL}_{i,h_2a_1} + f^{QTL}_{i,h_1a_1} f^{QTL}_{i,h_2a_2}) s^{\mathcal{P}}_{i,h_1,h_2} \\
&- s^{\mathcal{P}}_{i,h_2,h_2}(f^{QTL}_{i,h_2a_1} - f^{QTL}_{i,h_2a_2})^2 + ({f^{QTL}_{i,h_2a_1}}^2 + {f^{QTL}_{i,h_2a_2}}^2)
\end{aligned}
$$

and the frequencies in expression (2) can be written as:

$$
\begin{aligned}
f^{QTL}_{i,h_1a_1} &= f_{i,h_1} f_{a_1} + \mathbf{\Delta_1} = \alpha_1 + \mathbf{\Delta_1} \\
f^{QTL}_{i,h_1a_2} &= f_{i,h_1} f_{a_2} - \mathbf{\Delta_1} = \tilde{\alpha}_1 - \mathbf{\Delta_1} \\
f^{QTL}_{i,h_2a_1} &= f_{i,h_2} f_{a_1} - \mathbf{\Delta_1} = \alpha_2 - \mathbf{\Delta_1} \\
f^{QTL}_{i,h_2a_2} &= f_{i,h_2} f_{a_2} + \mathbf{\Delta_1} = \tilde{\alpha}_2 + \mathbf{\Delta_1}
\end{aligned}
$$

Note that $\mathbf{\Delta_1}$, in this case, can be expressed as $\mathbf{\Delta_1} = f^{QTL}_{i,h_1a_1} f^{QTL}_{i,h_2a_2} - f^{QTL}_{i,h_1a_2} f^{QTL}_{i,h_2a_1}$ with its maximum and minimum value given by $\frac{1}{4}$ and $-\frac{1}{4}$ respectively. The maximum value of $\mathbf{\Delta_1}$ is given by $f^{QTL}_{i,h_1a_1} =$

$f^{QTL}_{i,h_2a_2} = \dfrac{1}{2}$ and $f^{QTL}_{i,h_1a_2} = f^{QTL}_{i,h_2a_1} = 0$, and its minimum value is given by $f^{QTL}_{i,h_1a_1} = f^{QTL}_{i,h_2a_2} = 0$ and $f^{QTL}_{i,h_1a_2} = f^{QTL}_{i,h_2a_1} = \dfrac{1}{2}$. Replacing the haplotype frequencies in $d_1(\mathbf{M}^{\mathcal{P},i}, \mathbf{M}^{QTL})$ with the expressions including the $\alpha_1, \tilde{\alpha}_1, \tilde{\alpha}_2, \alpha_2$ and $\mathbf{\Delta_1}$ terms gives:

$$\begin{aligned}
d_1(\mathbf{M}^{\mathcal{P},i}, \mathbf{M}^{QTL}) &= \left[-4s^{\mathcal{P}}_{i,h_1,h_1} - 4s^{\mathcal{P}}_{i,h_2,h_2} + 8s^{\mathcal{P}}_{i,h_1,h_2}\right] \mathbf{\Delta_1}^2 \\
&+ \left[4s^{\mathcal{P}}_{i,h_1,h_1}(\tilde{\alpha}_1 - \alpha_1) + 4s^{\mathcal{P}}_{i,h_2,h_2}(\alpha_2 - \tilde{\alpha}_2) - 4s^{\mathcal{P}}_{i,h_1,h_2}(\tilde{\alpha}_1 + \alpha_2 - (\alpha_1 + \tilde{\alpha}_2))\right] \mathbf{\Delta_1} \\
&- s^{\mathcal{P}}_{i,h_1,h_1}(\tilde{\alpha}_1 - \alpha_1)^2 - s^{\mathcal{P}}_{i,h_2,h_2}(\alpha_2 - \tilde{\alpha}_2)^2 + 2s^{\mathcal{P}}_{i,h_1,h_2}(\tilde{\alpha}_1 - \alpha_1)(\alpha_2 - \tilde{\alpha}_2) \\
&+ (\alpha_1 + \alpha_2)^2 + (\tilde{\alpha}_2 + \tilde{\alpha}_1)^2 \\
&= \xi^{\mathcal{P}}(\mathbf{\Delta_1})
\end{aligned}$$

Hence $d_1(\mathbf{M}^{\mathcal{P},i}, \mathbf{M}^{QTL})$ can be expressed as:

$$\xi^{\mathcal{P}}(\mathbf{\Delta_1}) = \left[-4s^{\mathcal{P}}_{i,h_1,h_1} - 4s^{\mathcal{P}}_{i,h_2,h_2} + 8s^{\mathcal{P}}_{i,h_1,h_2}\right] \mathbf{\Delta_1^2} + \Psi^{\mathcal{P}} \mathbf{\Delta_1} + \Phi^{\mathcal{P}} \qquad (5)$$

For the extreme values of $\mathbf{\Delta_1}$ we have:

$$\xi^{\mathcal{P}}\left(\frac{1}{4}\right) = \xi^{\mathcal{P}}\left(-\frac{1}{4}\right) = \frac{1}{2} + \frac{1}{2}s^{\mathcal{P}}_{i,h_1,h_2} - \frac{1}{4}(s^{\mathcal{P}}_{i,h_1,h_1} + s^{\mathcal{P}}_{i,h_2,h_2})$$

This quantity can also be obtained simply from expression (2), when $K = 2$, by replacing f^{QTL}_{i,h_1a_1}, f^{QTL}_{i,h_2a_2}, f^{QTL}_{i,h_1a_2} and f^{QTL}_{i,h_2a_1} by their corresponding values for the maximum and minimum value of $\mathbf{\Delta_1}$. For $\mathbf{\Delta_1} = \dfrac{1}{4}$ we have:

$$\begin{aligned}
d_1(\mathbf{M}^{\mathcal{P},i}, \mathbf{M}^{QTL}) =& \left(\left(\frac{1}{2}\right)^2 + 0^2\right) - s^{\mathcal{P}}_{i,h_1,h_1}\left(\frac{1}{2} - 0\right)^2 + 2\left(\ \frac{1}{2}.0\ +\ 0.\frac{1}{2}\ \right)(1 - s^{\mathcal{P}}_{i,h_1,h_2}) \\
&+ 2\left(\ 0.0\ +\ \frac{1}{2}.\frac{1}{2}\ \right)s^{\mathcal{P}}_{i,h_1,h_2} - s^{\mathcal{P}}_{i,h_2,h_2}\left(0 - \frac{1}{2}\right)^2 + \left(0^2 + \left(\frac{1}{2}\right)^2\right) \\
=& \frac{1}{2} + \frac{1}{2}s^{\mathcal{P}}_{i,h_1,h_2} - \frac{1}{4}(s^{\mathcal{P}}_{i,h_1,h_1} + s^{\mathcal{P}}_{i,h_2,h_2}) \\
=& \xi^{\mathcal{P}}\left(\frac{1}{4}\right)
\end{aligned}$$

In the same manner we can show that $\xi^{\mathcal{P}}\left(-\frac{1}{4}\right) = \xi^{\mathcal{P}}\left(\frac{1}{4}\right)$ since the squares and the products of the frequencies in expression (2) when $K = 2$ are symmetric. $\frac{1}{2} + \frac{1}{2}s^{\mathcal{P}}_{i,h_1,h_2} - \frac{1}{4}(s^{\mathcal{P}}_{i,h_1,h_1} + s^{\mathcal{P}}_{i,h_2,h_2})$ is greater or equal to $\frac{1}{2}s^{\mathcal{P}}_{i,h_1,h_2}$ since the maximum possible value of $s^{\mathcal{P}}_{i,h_1,h_1}$ and $s^{\mathcal{P}}_{i,h_2,h_2}$ is equal to one. Hence if haplotypes h_1 and h_2 share allele similarity $s^{\mathcal{P}}_{i,h_1,h_2}$ will be positive and $\xi^{\mathcal{P}}\left(-\frac{1}{4}\right) = \xi^{\mathcal{P}}\left(\frac{1}{4}\right)$ $\in [\frac{1}{2}s^{\mathcal{P}}_{i,h_1,h_2}, 1]$. For $\mathcal{P} = \text{IBS}_{\text{hap}}$, i.e. $s^{\mathcal{P}}_{i,h_1,h_1} = s^{\mathcal{P}}_{i,h_2,h_2} = 1$ and $s^{\mathcal{P}}_{i,h_1,h_2} = 0$, we have $\xi^{\mathcal{P}}\left(-\frac{1}{4}\right) = \xi^{\mathcal{P}}\left(\frac{1}{4}\right) = 0$. Note that for $\mathcal{P} = \text{IBS}_{\text{hap}}$ $\xi^{\mathcal{P}}(\boldsymbol{\Delta_1})$ becomes:

$$\xi^{\text{IBS}_{\text{hap}}}(\boldsymbol{\Delta_1}) = -8\boldsymbol{\Delta_1}^2 + 4\left[(\tilde{\alpha}_1 - \alpha_1) + (\alpha_2 - \tilde{\alpha}_2)\right]\boldsymbol{\Delta_1} - (\tilde{\alpha}_1 - \alpha_1)^2 - (\alpha_2 - \tilde{\alpha}_2)^2 + (\alpha_1 + \alpha_2)^2 + (\tilde{\alpha}_2 + \tilde{\alpha}_1)^2 \qquad (6)$$

Thus differentiating $\xi^{\text{IBS}_{\text{hap}}}$ with respect to $\boldsymbol{\Delta_1}$ gives:

$$\boldsymbol{\Delta_1^*} = \frac{\tilde{\alpha}_1 - \alpha_1 + \alpha_2 - \tilde{\alpha}_2}{4} = \frac{(2f_{a_1} - 1)(1 - 2f_{i,h_1})}{4}$$

The minimum of $\boldsymbol{\Delta_1^*}$, which is equal to $-\frac{1}{4}$, is given by either $f_{a_1} = f_{i,h_1} = 1$ or $f_{a_1} = f_{i,h_1} = 0$. Its maximum value, which is equal to $\frac{1}{4}$, is given by either $f_{a_1} = 0$ and $f_{i,h_1} = 1$ or $f_{a_1} = 1$ and $f_{i,h_1} = 0$. Hence $\boldsymbol{\Delta_1^*}$ takes its minimum and maximum values when both the tested locus and the QTL are monomorphic.

Algebraic derivation of the matrix distance for a multiallelic QTL

For S distinct alleles at the QTL expression (2) generalizes to:

$$d_1(\mathbf{M}^{\mathcal{P},i}, \mathbf{M}^{QTL}) = \sum_{p=1}^{K}\sum_{q=1}^{K}\left[(1 - s^{\mathcal{P}}_{i,h_p,h_q})\sum_{l=1}^{S} f^{QTL}_{i,h_p a_l} f^{QTL}_{i,h_q a_l} + s^{\mathcal{P}}_{i,h_p,h_q}\sum_{l=1}^{S}\sum_{m\neq l}^{S} f^{QTL}_{i,h_p a_l} f^{QTL}_{i,h_q a_m}\right] \qquad (7)$$

For $\mathcal{P} = \text{IBS}_{\text{hap}}$ expression (7) becomes:

$$d_1(\mathbf{M}^{\mathrm{IBS_{hap}},i}, \mathbf{M}^{QTL}) = \sum_{p=1}^{K}\sum_{l=1}^{S}\sum_{m\neq l}^{S} f_{i,h_pa_l}^{QTL} f_{i,h_pa_m}^{QTL} + \sum_{p=1}^{K}\sum_{l=1}^{S}\sum_{q\neq p}^{K} f_{i,h_pa_l}^{QTL} f_{i,h_qa_l}^{QTL} \quad (7)$$

Let $\mathbf{\Delta_{pl}} = f_{i,h_pa_l}^{QTL} - f_{i,h_p}f_{a_l} = f_{i,h_pa_l}^{QTL} - \alpha_{pl}$, which is equivalent to $f_{i,h_pa_l}^{QTL} = \alpha_{pl} + \mathbf{\Delta_{pl}}$. Note that:

$$D_{i,QTL}^2 = \sum_{p=1}^{K}\sum_{l=1}^{S}(f_{i,h_pa_l}^{QTL} - f_{i,h_p}.f_{a_l})^2 = \sum_{p=1}^{K}\sum_{l=1}^{S}\mathbf{\Delta_{pl}}^2$$

Since $\sum_{p=1}^{K}\mathbf{\Delta_{pl}} = 0$ we have $\sum_{q\neq p}^{K}\mathbf{\Delta_{ql}} = -\mathbf{\Delta_{pl}}$ and $\sum_{m\neq l}^{S}\mathbf{\Delta_{pm}} = -\mathbf{\Delta_{pl}}$. Replacing the haplotype frequencies in expression (7) with the LD coefficients and the product of frequencies terms, and subsequently replacing $\sum_{q\neq p}^{K}\mathbf{\Delta_{ql}}$ and $\sum_{m\neq l}^{S}\mathbf{\Delta_{pm}}$ with $-\mathbf{\Delta_{pl}}$, gives:

$$\begin{aligned} d_1(\mathbf{M}^{\mathrm{IBS_{hap}},i}, \mathbf{M}^{QTL}) &= \sum_{p=1}^{K}\sum_{l=1}^{S}\Bigg[-2\mathbf{\Delta_{pl}^2} + \left(\Psi_{pl}^{\mathrm{IBS_{hap}},(1)} + \Psi_{pl}^{\mathrm{IBS_{hap}},(2)}\right)\mathbf{\Delta_{pl}} \\ &\qquad + \left(\Phi_{pl}^{\mathrm{IBS_{hap}},(1)} + \Phi_{pl}^{\mathrm{IBS_{hap}},(2)}\right)\Bigg] \\ &= \xi^{\mathrm{IBS_{hap}}}(\mathbf{\Delta_{11}}, \mathbf{\Delta_{12}},..., \mathbf{\Delta_{KS}}) \end{aligned}$$

As for expression (3) the general behavior of the matrix distance for continuous predictors in $[0,1]$, as function of LD coefficients, is unspecifiable for the multiallelic QTL case. Hence we did not express the matrix distance, here in the multiallelic QTL case, for continuous predictors in $[0,1]$.
