# Supplementary material for: Using haplotypes for the prediction of allelic identity to fine-map QTL: characterization and properties
Source: Genet Sel Evol. 2014 Jul 14;46(1):45. doi: 10.1186/1297-9686-46-45 (PMC4223544; doi:10.1186/1297-9686-46-45)
Supplement: Additional file 2 — Domains of LD coefficients and boundary conditions for the critical values of eachQp function. This file contains the domain of values for the multiallelic LD coefficients, the boundary conditions for the critical value of each Qp function in expression (4) and the relation between the sum of the squared deviations and Di,QTL2. [file 1297-9686-46-45-S2.pdf]

Additional file 2

Domains of LD coefficients and boundary conditions for the critical values of each Qp function

Domains of values for the multiallelic LD coefficients

Let $\mathbf{\Delta_p}$ be the LD coefficient between haplotype h_p and allele a_1 at the QTL, i.e.

$$\begin{cases} \mathbf{\Delta_p} = f^{QTL}_{i,h_p a_1} - f_{i,h_p} f_{a_1} \ (a) \\ -\mathbf{\Delta_p} = f^{QTL}_{i,h_p a_2} - f_{i,h_p} f_{a_2} \Leftrightarrow \mathbf{\Delta_p} = f_{i,h_p} f_{a_2} - f^{QTL}_{i,h_p a_2} \ (b) \end{cases} \qquad \begin{cases} f^{QTL}_{i,h_p a_1} = f_{i,h_p} f_{a_1} + \mathbf{\Delta_p} \ (c) \\ f^{QTL}_{i,h_p a_2} = f_{i,h_p} f_{a_2} - \mathbf{\Delta_p} \ (d) \end{cases}$$

$\mathbf{\Delta_p}$ is maximum in (a) and (b) when $f^{QTL}_{i,h_p a_1} = f_{a_1}$ and $f^{QTL}_{i,h_p a_2} = 0$ respectively. Under these conditions we also have $f_{i,h_p} = f^{QTL}_{i,h_p a_1} = f_{a_1}$ since $f_{i,h_p} = f^{QTL}_{i,h_p a_1} + f^{QTL}_{i,h_p a_2} = f_{a_1} + 0$. Hence $\mathbf{\Delta_p}$ can be written as $\mathbf{\Delta_p} = f_{i,h_p} - f^2_{i,h_p} = f_{i,h_p}(1 - f_{i,h_p})$ under these conditions. $f_{i,h_p}(1 - f_{i,h_p})$ is identifiable to the function $x \mapsto x(1-x)$ which takes a maximum value of $\frac{1}{4}$ for $x = \frac{1}{2}$. One of the maximum possible value for $\mathbf{\Delta_p}$ is thus given by $\frac{1}{4}$. In the same manner we can show that one of the minimum possible value for $\mathbf{\Delta_p}$ is given by $-\frac{1}{4}$. Since $f^{QTL}_{i,h_p a_1} \geq 0$ and $f^{QTL}_{i,h_p a_2} \geq 0$ we also have $\mathbf{\Delta_p} \geq -f_{i,h_p}.f_{a_1}$ and $\mathbf{\Delta_p} \leq f_{i,h_p}.f_{a_2}$ from (c) and (d) respectively. Hence the complete domain of values for each $\mathbf{\Delta_p}$ term is given by: $\mathbf{\Delta_p} \in \left[max(-\frac{1}{4}, -f_{i,h_p}.f_{a_1}), min(\frac{1}{4}, f_{i,h_p}.f_{a_2})\right]$.

Boundary conditions for the critical value of each Q_p function

Each Q_p function is given by:

$$Q_p(\mathbf{\Delta_p}) = -4\mathbf{\Delta_p^2} + \Psi^{\text{IBS}_\text{hap}}_{pq} \mathbf{\Delta_p} + \Phi^{\text{IBS}_\text{hap}}_{pq}$$

Differentiating Q_p with respect to $\mathbf{\Delta_p}$ gives $\mathbf{\Delta_p^*} = \frac{\Psi^{\text{IBS}_\text{hap}}_{pq}}{8}$ where $\Psi^{\text{IBS}_\text{hap}}_{pq}$ is given by:

$$\Psi_{pq}^{\text{IBS}_{\text{hap}}} = 3(\tilde{\alpha}_p - \alpha_p) + \sum_{q \neq p}^{K} (\alpha_q - \tilde{\alpha}_q)$$

See expression (3), with $s_{i,h_p,h_p}^{\mathcal{P}} = 1$ and $s_{i,h_p,h_q}^{\mathcal{P}} = 0$, in Additional file 1 for $\Psi_{pq}^{\text{IBS}_{\text{hap}}}$ and the corresponding products of frequencies for α_p and $\tilde{\alpha}_p$.

$$\begin{aligned}
\Psi_{pq}^{\text{IBS}_{\text{hap}}} &= 3(\tilde{\alpha}_p - \alpha_p) + \sum_{q \neq p}^{K} (\alpha_q - \tilde{\alpha}_q) \\
&= 3(\tilde{\alpha}_p - \alpha_p) + \sum_{q \neq p}^{K} (\alpha_q - \tilde{\alpha}_q) + \alpha_p - \tilde{\alpha}_p - (\alpha_p - \tilde{\alpha}_p) \\
&= 4(\tilde{\alpha}_p - \alpha_p) + \sum_{q=1}^{K} (\alpha_q - \tilde{\alpha}_q) \\
&= 4 f_{i,h_p}(f_{a_2} - f_{a_1}) + (f_{a_1} - f_{a_2}) \sum_{q=1}^{K} f_{i,h_q} = (f_{a_1} - f_{a_2})[1 - 4 f_{i,h_p}]
\end{aligned}$$

Hence we have $\mathbf{\Delta_p^*} = \frac{(f_{a_1} - f_{a_2})[1 - 4 f_{i,h_p}]}{8} = \frac{(2 f_{a_1} - 1)[1 - 4 f_{i,h_p}]}{8}$. Let $\mathbf{\Delta_{p_{min}}} = max(-\frac{1}{4}, -f_{i,h_p}.f_{a_1})$ and $\mathbf{\Delta_{p_{max}}} = min(\frac{1}{4}, f_{i,h_p}.f_{a_2})$. Note that $0 \in \left]\mathbf{\Delta_{p_{min}}}, \mathbf{\Delta_{p_{max}}}\right[$. Hence if $\mathbf{\Delta_p^*} \in \left]\mathbf{\Delta_{p_{min}}}, \mathbf{\Delta_{p_{max}}}\right[$ and the magnitude (absolute value) of $\mathbf{\Delta_p}$ increases sufficiently Q_p will decrease. Figure 8 gives an example of a Q_p function with $\mathbf{\Delta_p^*} \in \left]\mathbf{\Delta_{p_{min}}}, \mathbf{\Delta_{p_{max}}}\right[$.

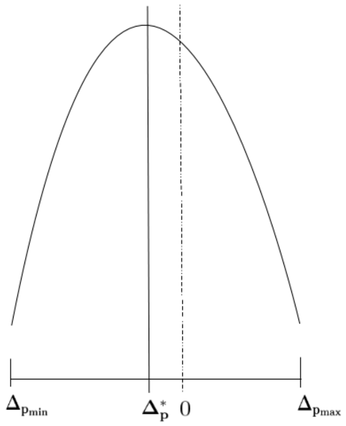

FIGURE 8: An example of a Q_p function with $\mathbf{\Delta_p^*} \in \left]\mathbf{\Delta_{p_{min}}}, \mathbf{\Delta_{p_{max}}}\right[$.

The only situations for which it is not possible to tell if Q_p will decrease, as the magnitude of $\mathbf{\Delta_p}$ increases sufficiently, are given by the following conditions; $\mathbf{\Delta_p^*} \leq \mathbf{\Delta_{p_{min}}}$ (e) or $\mathbf{\Delta_p^*} \geq \mathbf{\Delta_{p_{max}}}$ (f). In these situations Q_p can either decrease or increase if the magnitude of $\mathbf{\Delta_p}$ increases sufficiently. Figure 9 gives an example of a Q_p function with $\mathbf{\Delta_p^*} \leq \mathbf{\Delta_{p_{min}}}$.

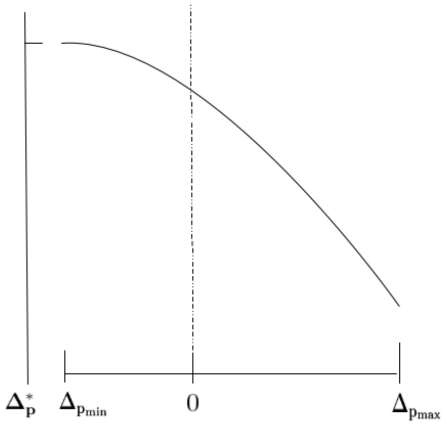

FIGURE 9: An example of a Q_p function with $\mathbf{\Delta_p^*} \leq \mathbf{\Delta_{p_{min}}}$.

Conditions (e) and (f) can be written as follows:

$$(e)\begin{cases} \text{If } \mathbf{\Delta_{p_{min}}} = -\dfrac{1}{4} \text{ then } \mathbf{\Delta_p^*} \leq \mathbf{\Delta_{p_{min}}} \Leftrightarrow (2f_{a_1} - 1)[1 - 4f_{i,h_p}] \leq -2 \\ \text{or} \\ \text{If } \mathbf{\Delta_{p_{min}}} = -f_{i,h_p}.f_{a_1} \text{ then } \mathbf{\Delta_p^*} \leq \mathbf{\Delta_{p_{min}}} \Leftrightarrow \dfrac{(2f_{a_1} - 1)[1 - 4f_{i,h_p}]}{f_{i,h_p}.f_{a_1}} \leq -8 \end{cases}$$

$$(f)\begin{cases} \text{If } \mathbf{\Delta_{p_{max}}} = \dfrac{1}{4} \text{ then } \mathbf{\Delta_p^*} \geq \mathbf{\Delta_{p_{max}}} \Leftrightarrow (2f_{a_1} - 1)[1 - 4f_{i,h_p}] \geq 2 \\ \text{or} \\ \text{If } \mathbf{\Delta_{p_{max}}} = f_{i,h_p}(1 - f_{a_1}) \text{ then } \mathbf{\Delta_p^*} \geq \mathbf{\Delta_{p_{max}}} \Leftrightarrow \dfrac{(2f_{a_1} - 1)[1 - 4f_{i,h_p}]}{f_{i,h_p}(1 - f_{a_1})} \geq 8 \end{cases}$$

Let w, t, s and u be the following functions of f_{a_1} and f_{i,h_p}; $w(f_{a_1}, f_{i,h_p}) = (2f_{a_1}-1)[1-4f_{i,h_p}]+2$, $t(f_{a_1}, f_{i,h_p}) = \dfrac{(2f_{a_1}-1)[1-4f_{i,h_p}]}{f_{i,h_p}.f_{a_1}} + 8$, $s(f_{a_1}, f_{i,h_p}) = (2f_{a_1}-1)[1-4f_{i,h_p}] - 2$ and $u(f_{a_1}, f_{i,h_p}) = \dfrac{(2f_{a_1}-1)[1-4f_{i,h_p}]}{f_{i,h_p}(1-f_{a_1})} - 8$. Conditions (e) and (f) are the same as searching values of f_{a_1} and f_{i,h_p} for which we have:

$$(e)\begin{cases} w \leq 0 \\ \text{or} \\ t \leq 0 \end{cases} \qquad (f)\begin{cases} s \geq 0 \\ \text{or} \\ u \geq 0 \end{cases}$$

Figure 10 shows the regions (red colored), for different a_1 and h_p frequencies, where condition (e) or (f) is realized. As can be seen in figure 10, the conditions for w, t, s and u are verified when f_{a_1} and f_{i,h_p} are both high or both low, or one of these two frequencies is high and the other one is low. Note that these frequencies correspond to situations where Q_p can still decrease as suggested by figure 11 (see relation between the sum of the squared deviations and $D^2_{i,QTL}$).

Moreover these frequencies correspond to situations which are unfavorable for QTL analysis as low frequencies do not allow for reliable estimation and comparison of contrasts between groups of individuals. Finally note that LD requires variation of alleles between loci to exist. Hence these high or low frequencies correspond to situations which are unfavorable for LD mapping of QTL.

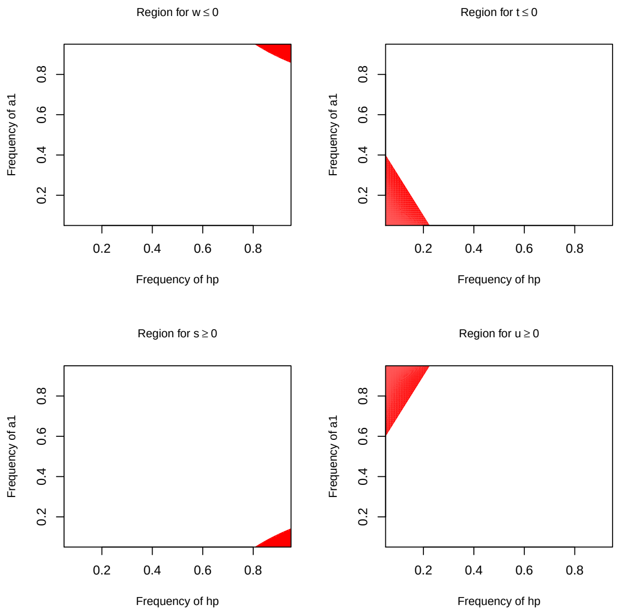

FIGURE 10: The different regions (red colored) where condition (e) or (f) is realized.

Relation between the sum of the squared deviations and $D^2_{i,QTL}$

Let $D^2_{i,QTL} = 2\sum_{p=1}^{K} \mathbf{\Delta_p^2}$ be the non-normalized multiallelic measure of LD and $SD_{i,QTL} = \sum_{p=1}^{K}(\mathbf{\Delta_p} - \mathbf{\Delta_p^*})^2$ be the sum of the squared deviations of the multiallelic LD coefficients from their corresponding $\mathbf{\Delta_p^*}$ critical values. $SD_{i,QTL}$ can be written as a sum of convex U_p functions of each LD coefficient, i.e.

$$SD_{i,QTL} = \sum_{p=1}^{K}(\mathbf{\Delta_p} - \mathbf{\Delta_p^*})^2 = \sum_{p=1}^{K} \mathbf{\Delta_p^2} - \omega\mathbf{\Delta_p} + \upsilon = \sum_{p=1}^{K} U_p(\mathbf{\Delta_p})$$

where $\omega = 2\mathbf{\Delta_p^*}$ and $\upsilon = \mathbf{\Delta_p^*}^2$, and the critical value of each U_p function is $\mathbf{\Delta_p^*}$. Hence there is an implicit relationship between $D^2_{i,QTL}$ and $SD_{i,QTL}$. If the sum of the squared $\mathbf{\Delta_p}$ terms increases sufficiently (i.e. $\frac{D^2_{i,QTL}}{2}$ increases sufficiently) $SD_{i,QTL}$ will increase. The same procedure as the one used to describe the expected behavior of $D^2_{i,QTL}$ was repeated for $SD_{i,QTL}$ and $\frac{D^2_{i,QTL}}{2}$ on the 889 FLW regions (see variation of LD subsection in methods). That is both $\mathbf{\Delta_p}$ and $\mathbf{\Delta_p^*}$ were computed at

each tested position, in order to compute $SD_{i,QTL}$ and $\frac{D^2_{i,QTL}}{2}$, while screening the 889 regions. Figure 11 shows the profiles of the empirical means of the 889 FLW curves for $SD_{i,QTL}$ and $\frac{D^2_{i,QTL}}{2}$, and the deviation $\left(\mathbb{E}[SD_{i,QTL} - \frac{D^2_{i,QTL}}{2}] = \mathbb{E}[-\omega\boldsymbol{\Delta}_{\mathbf{p}} + \upsilon]\right)$ between these two profiles. As observed in figure 11, the profiles for the expected values of $SD_{i,QTL}$ and $\frac{D^2_{i,QTL}}{2}$ exhibit similar patterns with a relatively small increasing deviation, between these two profiles, as the tested position moves toward the QTL. Note that the profile for the deviation exhibit a similar trend to the profiles for the expected values of $SD_{i,QTL}$ and $\frac{D^2_{i,QTL}}{2}$.

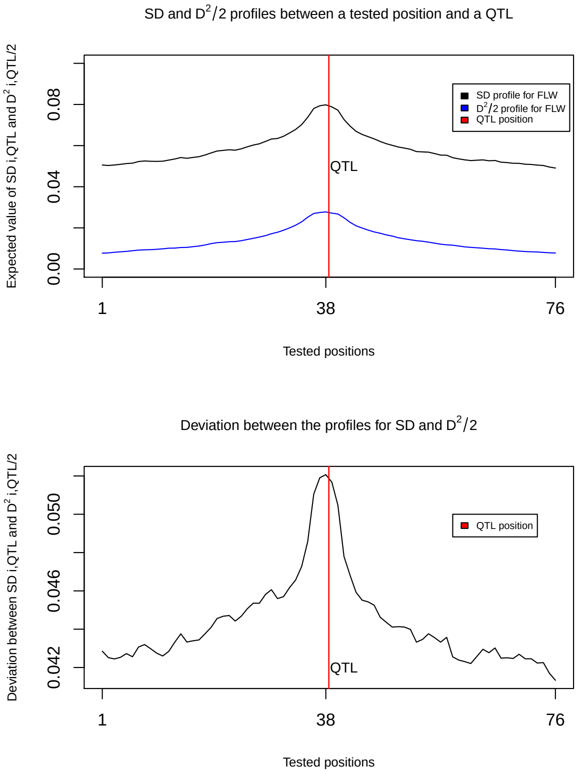

FIGURE 11: Empirical means of the 889 FLW curves, obtained for $D^2_{i,QTL}$ and $SD_{i,QTL}$ between tested positions (tested position i = center of 6 marker haplotypes) and a biallelic QTL (red vertical line) for regions of 81 markers on chromosomes, and the deviation between the mean curves.
